# The central amygdala recruits mesocorticolimbic circuitry for pursuit of reward or pain

**DOI:** 10.1038/s41467-020-16407-1

**Published:** 2020-06-01

**Authors:** Shelley M. Warlow, Erin E. Naffziger, Kent C. Berridge

**Affiliations:** 1 Department of Psychology, University of Michigan, 530 Church St., Ann Arbor, MI 48109 USA; 2 Present Address: Department of Neurosciences, University of California San Diego, La Jolla, CA 92093 USA

**Keywords:** Motivation, Reward

## Abstract

How do brain mechanisms create maladaptive attractions? Here intense maladaptive attractions are created in laboratory rats by pairing optogenetic channelrhodopsin (ChR2) stimulation of central nucleus of amygdala (CeA) in rats with encountering either sucrose, cocaine, or a painful shock-delivering object. We find that pairings make the respective rats pursue either sucrose exclusively, or cocaine exclusively, or repeatedly self-inflict shocks. CeA-induced maladaptive attractions, even to the painful shock-rod, recruit mesocorticolimbic incentive-related circuitry. Shock-associated cues also gain positive incentive value and are pursued. Yet the motivational effects of paired CeA stimulation can be reversed to negative valence in a Pavlovian fear learning situation, where CeA ChR2 pairing increases defensive reactions. Finally, CeA ChR2 valence can be switched to neutral by pairing with innocuous stimuli. These results reveal valence plasticity and multiple modes for motivation via mesocorticolimbic circuitry under the control of CeA activation.

## Abstract (web summary)

Brain disorders can create maladaptive attractions, such as in addiction or self-harming. Here the authors use multiple valence modes of the central amygdala to create such attractions, arbitrarily making rats into ‘sucrose addicts' or ‘cocaine addicts', or causing maladaptive attraction to shocks.

## Introduction

The amygdala and related mesocorticolimbic circuitry help assign motivational significance to both reward-related and threat-related stimuli^1–6^. In clinical disorders, maladaptive attractions can become intense and narrowly focused on inappropriate targets, as in addictions and self-harming^7,8^. Here we explore amygdala-triggered mechanisms that induce maladaptive attractions, operationally defined as attractions that are excessively intense (e.g., more than double the attraction than the same target supports in ordinary individuals), simultaneously narrowly focused (e.g., attraction pulled nearly entirely to one target, among otherwise equally attractive targets), and which carry adverse consequences (e.g., pain).

Intense and narrowly focused attractions have been induced in laboratory rats by pairing of optogenetic channelrhodopsin (ChR2) stimulation of neurons in central nucleus of amygdala (CeA) with sensory rewards, intensifying appetitive motivation (e.g., effort breakpoints) and narrowing pursuit to the paired reward in choice tasks^9–11^. We report here that CeA ChR2 pairings can further narrow attraction at experimenter’s whim to either a natural sucrose reward or cocaine drug reward when both rewards are available, thus arbitrarily making a rat into either a ‘sucrose addict’ that ignores alternative intravenous cocaine, or conversely a ‘cocaine addict’ that ignores sucrose. Further, CeA ChR2 pairing can create maladaptive attraction to a noxious stimulus, such as an electrified shock rod, which normally elicits avoidance and fear-related defensive reactions^12,13^. The value of motivation produced by CeA ChR2 pairings can also switch to negative valence in a traditional Pavlovian fear learning context, oppositely increasing conditioned defensive reactions to cues for the uncontrollable footshock. Finally, CeA ChR2 valence can further switch to relatively neutral when laser is delivered by itself or paired with innocuous stimuli. Thus, CeA ChR2 control of mesocorticolimbic circuitry can create either maladaptive attractions, exaggerated fear reactions, or become relatively neutral by interacting with situational factors.

## Results

### CeA ChR2 virus and Fos protein expression

**Fig. 1:**
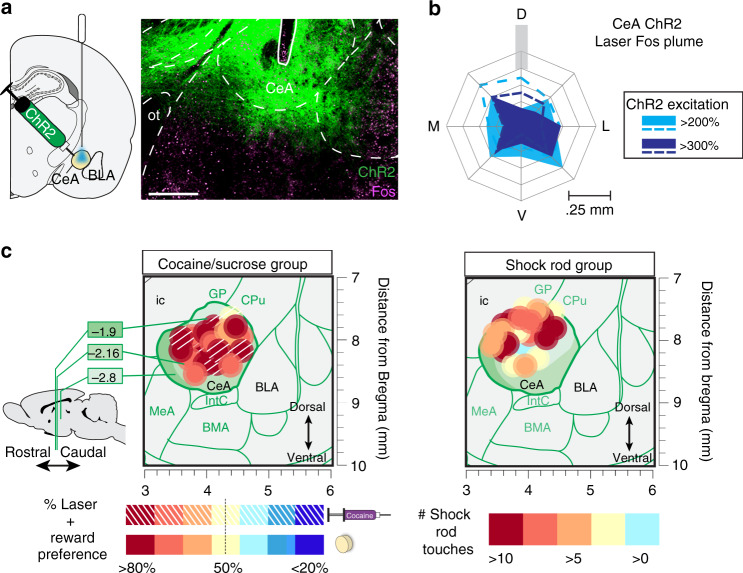
CeA ChR2 virus and Fos plumes. **a** CeA photomicrograph (×10 magnification) shows green channelrhodopsin (ChR2) virus infection (AAV5-hSyn-ChR2-eYFP) and magenta Fos protein (*N* = 16 rats; ot: optic tract; scale bar: 0.5 mm). **b** Average Fos plume in CeA-mapped ChR2 rats after laser illumination (>200% above mean eYFP control baseline: light solid blue, >200% above normal unoperated tissue baseline: light blue dashed lines; >300% above eYFP: dark solid blue, >300% above baseline: dark blue dashed lines; D: dorsal, M: medial, L: lateral, V: ventral). See also Supplementary Fig. 1. **c** Mapped CeA sites of optic fiber implants for each ChR2 rat in cocaine/sucrose group and in shock-rod group (coronal view). Size of each site symbol reflects size of Fos plumes. Symbol colors in sucrose–cocaine group represents the percentage preference for individual’s laser-paired sucrose/cocaine reward over alternative reward. For Shock-Rod Group, symbol color represents individual’s number of shock-rod touches on day 3. Ic, internal capsule; GP, globus pallidus; CPu, caudate putamen; BLA, basolateral amygdala; IntC, intercalated amygdala; MeA, medial amygdala; BMA, basomedial amygdala.

Laser stimulation of ChR2-infected CeA neurons produced local zones of excitation in CeA reflected in local Fos plumes of 0.15–0.2 mm radius around optic fiber tips. Plumes contained >200–300% elevations in Fos, compared with baseline levels in eYFP or unoperated controls (Fig. 1a, b and Supplementary Fig. 1). Fos plume diameters were used to determine the size of 0.4 mm placement symbols for functional maps in figures (Fig. 1c, d). CeA ChR2 laser pairings also activated distant brain circuitry as described below.

### Sucrose vs. cocaine two-choice task

**Fig. 2:**
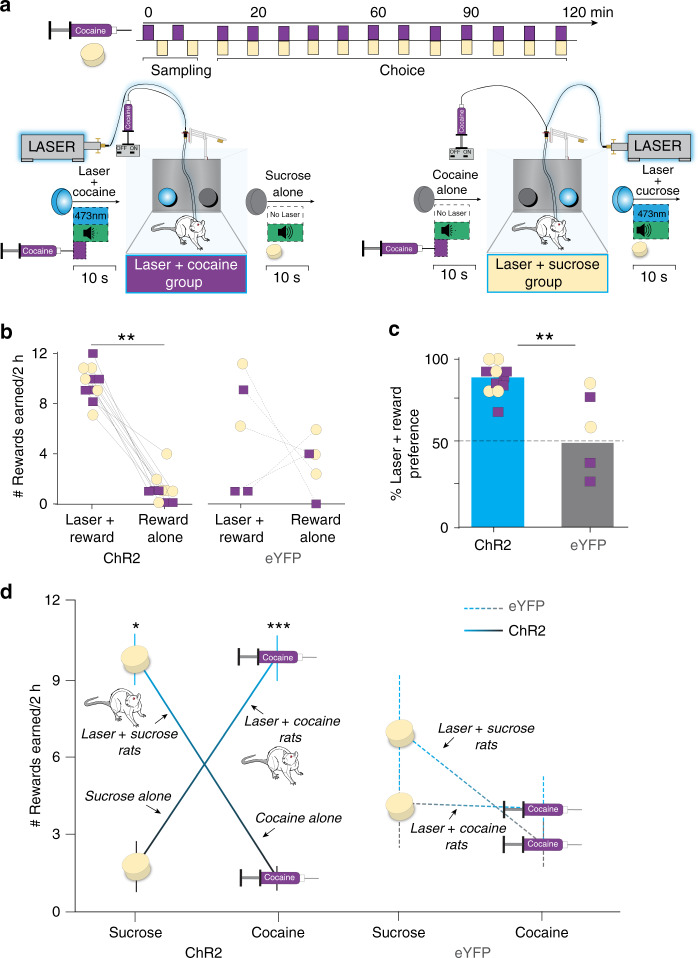
CeA ChR2 pairing controls pursuit of sucrose vs. cocaine. **a** Sucrose–cocaine choice paradigm and timeline within an individual test session; nm, nanometers; s, seconds; min, minutes. **b** Earned rewards (via nosepokes on an FR1 schedule) during 2 h (h) sucrose vs. cocaine choice sessions. ChR2 rats, *N* = 11; eYFP rats, *N* = 5; two-way within and between subjects ANOVA, main effect of virus: *F*_1,10_ = 4.6, *p* = 0.046; ChR2 laser vs. nonlaser responses, two-sided paired *t*-test, *t*_9_ = 7.5, *p* = 0.000, 95% CI: 4.8, 9.2, *d* = 4.39. Individuals with laser-paired sucrose in yellow circles and cocaine in purple squares. **c** Percent preference for laser-paired reward on day 4 (ChR2 rats: *N* = 11; eYFP rats: *N* = 5; two-sided unpaired *t*-test, *t*_10_ = 3.4, *p* = 0.006, 95% CI: 13, 60, *d* = 1.81). **d** Number of earned rewards for sucrose reward versus cocaine reward on last choice session, separated by reward type. CeA ChR2 laser + sucrose: *N* = 5 rats, sucrose vs. cocaine alone: two-sided paired *t*-test, *t*_3_ = 4.72, *p* = 0.04, 95% CI: 1, 15, Cohen’s *d* = 5.11; CeA ChR2 laser + cocaine: *N* = 6 rats, sucrose alone vs. cocaine: two-sided paired *t*-test, *t*_5_ = 7.7, *p* = 0.000, 95% CI: 5.3, 9.2, *d* = 4.6). Control eYFP laser  +  sucrose: *N* = 2 rats, eYFP laser + cocaine: *N* = 3 rats; two-way repeated measures ANOVA, main effect of laser: *F*_1,3_ = 1.35, *p* = 0.33; h, hour. All data represent means and SEM. *p < 0.05, **p < 0.01, ***p < 0.001.

We first assessed the effect of pairing CeA ChR2 stimulations with earning either sucrose or cocaine in an instrumental nose-poke task, when rats were given choices between the two rewards^14,15^ (Fig. 2a). Control rats with optically inactive virus in CeA (‘eYFP’) chose about equally between sucrose and cocaine regardless of which was paired with laser (Fig. 2b, c). By contrast, CeA ChR2 rats with amygdala laser stimulation paired with sucrose continually pursued and consumed only sucrose, ignoring cocaine. Conversely, different CeA ChR2 rats with laser stimulation paired with cocaine, exclusively pursued cocaine, while ignoring sucrose (both 87 + 4% preference by day 4, or a 10:2 ratio, compared with eYFP 1:1 ratio of 49 + 13%; Fig. 2d; also see Supplementary Fig. 2a, b*;* Supplementary Movie 1).

CeA ChR2 rats were also >30 times faster to initiate nosepokes into their laser-paired porthole than into their nonlaser porthole, once each was available on single-choice trials (within 3 ± 0.3 s; median: 2), regardless of whether their laser-paired porthole earned sucrose or cocaine (97 ± 12 s; median: 40) (*N* = 11, Wilcoxon signed-ranks test: *Z* = 2.8, *p* = 0.005). After earning a reward, CeA ChR2 rats continued to perseverate in making repeated additional nosepokes specifically in their laser-paired porthole during the ensuing 8-s time-out period (5 ± 1 total perseverative pokes), despite perseverative responses earning nothing (Supplementary Fig. 2c).

CeA ChR2 pairing also increased consummatory actions targeted toward associated metal cues. CeA ChR2 rats nibbled and bit their laser-paired porthole twice as much as they nibbled/bit the nonlaser porthole regardless of whether the laser-paired reward was cocaine or sucrose (Supplementary Fig. 2d). Increases in consummatory actions directed toward Pavlovian cues for reward is a sign of heightened incentive salience, which can make cues become perceived as more orally attractive and consumable^6,10,16,17^.

### CeA ChR2 pairing creates attraction to noxious shock rod

**Fig. 3:**
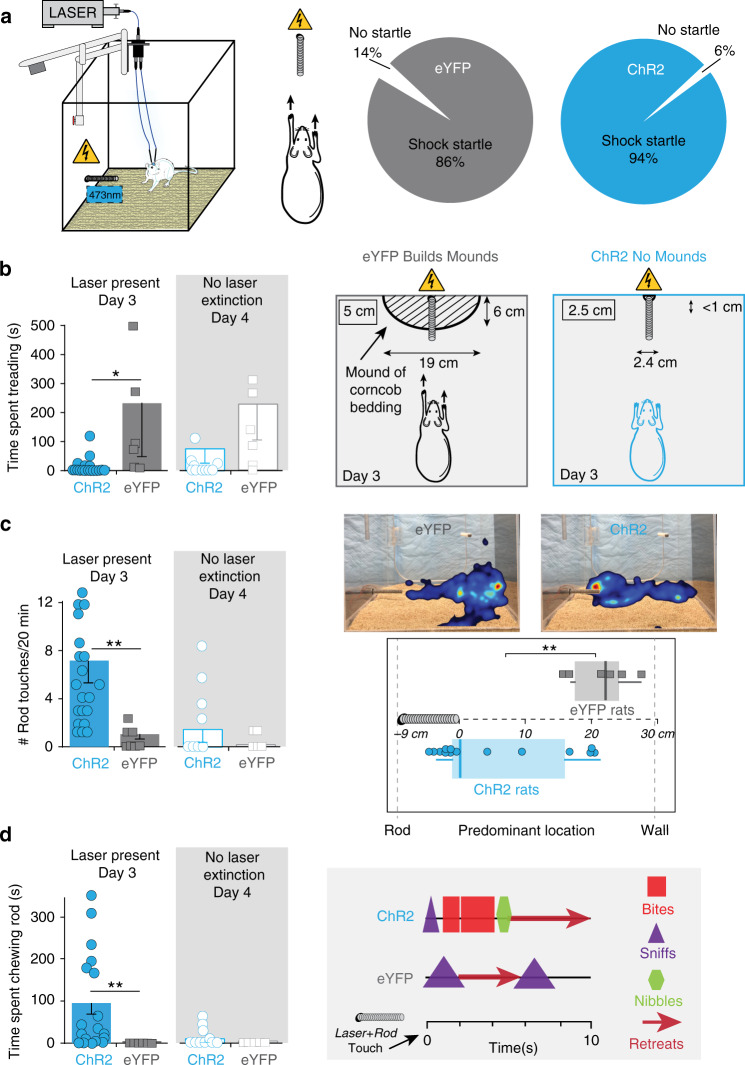
CeA paired stimulation creates attraction toward aversive shock rod. **a** Apparatus: electrified shock rod protruded ~9 cm out from wall into plexiglas chamber. CeA laser illumination was paired with shock-rod encounters (when rat was within <2-cm proximity). Startle probability (i.e., paw withdrawal or jump) when touching the shock rod during the first encounter (ChR2, *N* = 16 vs. eYFP, *N* = 6 two-sided unpaired *t*-test: *t*_30_ = 0.823, *p* = 0.42). **b** Defensive treading on day 3 when laser was present (CeA ChR2 *N* = 16 vs. eYFP rats *N* = *6*, two-sided unpaired *t*-test: *t*_21_ = −2.4, *p* = 0.03, 95% CI: 29, 410, *d* = 0.72) versus when laser was removed on day 4 (‘No Laser Extinction’, two-sided unpaired *t*-test: *t*_14_ = −1.69, *p* = 0.16). Drawing depicts defensive treading–burying behavior and consequent mounds of bedding. Placement and size of mounds shown by diameter of striped mound symbol (ChR2 vs. eYFP two-sided unpaired t-tests: height: *t*_26_ = −4.3, *p* = 0.000, 95% CI: −3.5, −1.2, *d* = 1.73; length: *t*_26_ = -5.4, *p* = .000, 95% CI: −6.9, −3.6, *d* = 2.09; width: *t*_26_ = −6.5, *p* = 0.000, 95% CI: −22.2, −11.6, *d* = 2.34). **c** Shock-rod touches when laser was paired with rod encounters (ChR2 rats, *N* = 16 vs. eYFP rats, *N* = 6, two-sided unpaired *t*-test: *t*_23_ = 3.6, *p* = 0.002, 95% CI: −11.8, −3.2, 11, *d* = 1.07), versus on no-laser day (two-sided unpaired *t*-test: *t*_11_ = 1.18, *p* = 0.26). Representative heatmaps (right) for individual rats shows location of representative rats during 20-min session (ChR2 or eYFP). Boxplots show predominant location: middle lines depict median (center), outer left lines extend to minimum value, outer right lines extend to maximum, and bounds of each box depict quartiles 2–3 (middle 50% of data; ChR2 rats, *N* = 14 vs. eYFP rats, *N* = 6, two-sided unpaired *t*-test: *t*_18_ = −3.8, *p* = 0.001, 95% CI: −25, −7.2, *d* = 2.11). **d** Time spent chewing shock rod with laser present (ChR2 rats, *N* = 16, eYFP rats, *N* = 6, two-sided unpaired *t*-test: *t*_18_ = −3.3, *p* = 0.004, 95% CI: −140, −30, *d* = 1.06) versus when laser was removed (two-sided unpaired *t*-test: *t*_11_ = 0.9, *p* = 0.38). Right panel shows microstructure choreograph of consummatory behaviors toward the shock rod from a representative ChR2 rat (top) and eYFP rat (bottom) during 10-s period following a shock rod touch. All data represent means and SEM. cm, centimeters. *p < 0.05, **p < 0.01.

In a different situation with a noxious shock rod, separate groups of CeA ChR2 and eYFP rats received pairings of CeA laser each time they voluntarily approached within 2 cm of the electrified rod (laser 40 Hz; 10 mW; bin duration 1–8 s, depending on how long the rat remained within 2-cm proximity of shock rod; shock rod = 1 cm in diameter and 9-cm long, wrapped with electrified wire that delivered 0.2–0.5-mA shock, depending on <0.25- to >1-s duration of contact) (Fig. 3a).

Control eYFP rats quickly learned to avoid the shock rod after touching it once or twice, and remained as far as possible from the rod for the remainder of the 20-min session. eYFP rats often emitted an active species-specific antipredator reaction of defensive treading–burying directed toward the shock rod (Fig. 3a)^12,13^. This often resulted in a small mound of cob bedding gradually being built around the rod during the 20-min session (Fig. 3b). Eighty-eight percent of eYFP rats emitted defensive treading/burying bouts longer than 10-s duration, and 75% of eYFP rats did so even on the first day (Supplementary Fig. 3a).

By contrast, CeA ChR2 rats approached and touched the rod five times on average the first day, receiving five shocks, touched and received seven shocks on the second day, and touched and received eight shocks on the third day (Fig. 3c; Supplementary Fig. 3b). Upon each shock, CeA ChR2 rats reacted immediately with reflexive startle and withdrawal reactions, just as eYFP control rats did, suggesting electric shock retained aversive impact during laser stimulation (Fig. 3a; Supplementary Movie 2). But after receiving a shock, CeA ChR2 rats typically returned within seconds or minutes to the rod, continually hovering closely over it, and soon received another shock to paw or mouth (Fig. 3c). Only one CeA ChR2 rat emitted any defensive treading–burying bout of longer than 10-s duration on the 3 days, and that rat did so only once (36% of CeA ChR2 rats never showed any antipredator behavior at all on any day; Fig. 3b).

### Consummatory chewing and sniffs of rod

CeA ChR2 rats additionally emitted occasional consummatory actions of chewing, nibbling, or biting on the metal shock rod during bouts of continuous rod sniffing. At least 66% (14/21) of CeA ChR2 rats nibbled or chewed the rod at least once on the first day, 71% (15/21) on the second day, and 66% (14/21) on the third day, on which they spent an average cumulative duration of 81 ± 24 s of oral nibbling or chewing on the shock rod (e.g., 5% of a 20-min session; Fig. 3c, Supplementary Fig. 3d). Consequently, CeA ChR2 rats often incurred shocks directly on their mouth, tongue or teeth, or on their nose while sniffing too closely (0.2 mA intensity within 0.5 s of chewing, reaching 0.4–0.5 mA within 2–3 s according to ammeter readings). A few chewing bouts reached up to 10–20-s duration (typically composed of several 2–4 s continual chewing bouts separated by brief <1 s withdraws/pauses). By contrast, no eYFP control rat ever nibbled or bit the rod on any day (Fig. 3d and Supplementary Fig. 3c).

Oral consummatory actions were likely not a simple motor effect of CeA ChR2 activation. The same rats failed to increase chewing of an inedible wooden block paired with ChR2 stimulation in separate tests, and there was no individual correlation between duration of shock-rod chewing and wooden block chewing (Pearson correlation, *r* = 0.049, *p* = 0.92). Similarly, CeA ChR2 laser stimulation did not cause greater touches of the wooden block (Supplementary Fig. 4b). In a separate test, laser pairings also failed to induce attraction to a dummy “no-shock rod” that was nearly identical to shock rod but was unelectrified. CeA ChR2 rats never chewed on the dummy rod, and touched the dummy rod no more often than eYFP controls (Supplementary Fig. 4a).

CeA ChR2 attraction to shock rod, once established, appeared robust across a range of optogenetic laser frequency and intensity parameters. Subsequent tests with paired laser frequencies of either 10, 25, or 40 Hz at 10 mW intensity, or constant illumination of laser at 1 mW intensity (constant low illumination is thought to facilitate endogenous firing patterns, rather than impose an artificial firing frequency^18^), all produced similar levels of shock-rod attraction and chewing in CeA ChR2 rats as the original 40 Hz 10 mW laser stimulation (Supplementary Fig. 5), suggesting CeA ChR2 attraction does not depend on any particular single laser parameter.

To assess if shock-rod attraction required concomitant CeA stimulation, rats were re-exposed to the electrified shock rod on a separate ‘laser extinction’ day, during which CeA laser illumination was no longer administered. All CeA ChR2 rats initially approached and touched the rod at least once, but ceased chewing the rod almost entirely after receiving the first shock, and 7/8 ChR2 rats reduced rod approaches and touches to less than half their number on the previous day when laser had last been delivered (Fig. 3c). Further, ChR2 rats also began to emit short bouts of defensive treading/burying (averaging ~5 s) toward the rod for the first time (Fig. 3b). We conclude CeA ChR2 rats remain able to recognize the noxious qualities of shock rod, and their full level of attraction to the shock rod is not simply due to a permanent learned re-evaluation but also depends in part on simultaneous rod-paired CeA ChR2 stimulations during the session.

### Motivated rod attraction overcomes obstacle

**Fig. 4:**
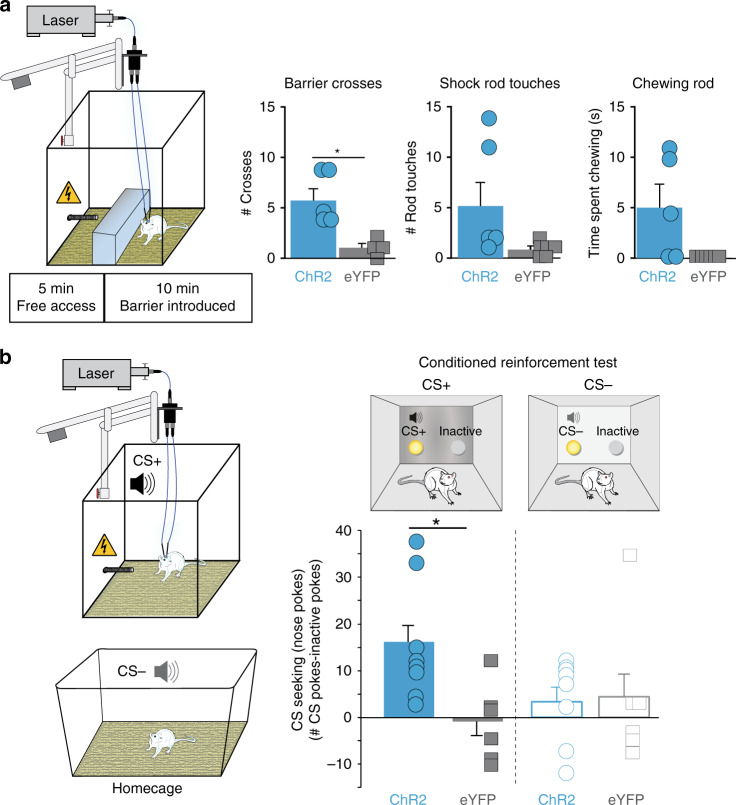
CeA ChR2 shock-rod attraction enhances shock-rod ‘seeking’ and cue incentive salience. **a** Apparatus shows novel opaque barrier interposed between rat and shock rod after 5 min into session. Number of barrier crosses to reach shock rod (ChR2 rats, *N* = 5 vs. eYFP rats, *N* = 5, two-sided unpaired *t*-test: *t*_8_ = 3.0, *p* = 0.02, *d* = 2.04). Number of shocked touches on shock rod: two-sided unpaired *t*-test: *t*_8_ = 1.1, *p* = 0.08. Time spent chewing on shock rod: two-sided unpaired *t*-test: *t*_8_ = 1.1, *p* = 0.08. **b** Instrumental conditioned reinforcement test. Rats nosepoked to earn presentations of auditory conditioned stimulus (CS)+ previously paired with shock-rod encounters (top) or a different auditory CS− previously paired with homecage. Right graph depicts CS+ seeking as difference score between the number of nosepokes to earn CS+ sound (shock-rod-paired CS) over the number of nosepokes in inactive hole that earned nothing. Left depicts CS− seeking: the number of nosepokes to earn CS− (homecage sound) sound over the number of nosepokes in inactive hole. CS+ vs. CS− were tested in separate sessions. Difference score for each day (CS nosepokes − inactive nosepokes) during CS+ and CS− sessions (ChR2 rats, *N* = 8, eYFP rats, *N* = 6*;* two-way repeated measures ANOVA, CS type × virus interaction: *F*_1,12_ = 3.84, *p* = 0.04; CS+: two-sided unpaired *t*-test: *p* = 0.03, 95% CI: 1.5, 29.6, *d* = 1.35; CS−: two-sided unpaired *t*-test: *p* = 0.57). Data represent mean and SEM. *p < 0.05.

We next assessed if CeA ChR2 rats were motivated to overcome an obstacle to reach the shock rod, when it was not immediately perceived. A large opaque obstacle block was interposed between the rat and the shock rod early in a session. The block completely filled the width of the chamber, prevented easy viewing of the rod, and required the rat to climb over it in order to reach the rod (Fig. 4a). All ChR2 rats actively climbed over the block (5/5) to touch the shock rod, upon which they were returned to the other side of the barrier. CeA ChR2 rats persisted in repeatedly climbing over the block 5 ± 1 times and received 3.36 ± 1 shocks per 15 min session, compared with eYFP rats that made 0 or 1 crosses, and did not receive shocks. CeA ChR2 rats also still typically chewed on the shock rod once they reached it (>5-s bouts), whereas eYFP rats never chewed (Fig. 4a).

### CeA ChR2 rats ‘want’ shock-associated cues

Given that incentive salience typically makes Pavlovian reward cues become ‘wanted’ themselves^19^, we assessed whether shock-rod cues gained their own incentive value by asking whether CeA ChR2 rats that had been attracted to shock rod would ‘want’ to hear an auditory Pavlovian cue associated with shocks (distinctive auditory tone or white noise presented during rod encounters and CeA laser stimulations; counterbalanced across rats). In an instrumental conditioned reinforcement test, conducted in novel chambers with shock-rod absent (Fig. 4b), rats were given the opportunity to nosepoke to earn 4-s presentations of either the shock-associated auditory cue (CS+) on 1 day, or an alternative auditory cue that had been heard in their homecage (CS−) on a separate day. During both days, nosepokes into another porthole earned nothing, serving as a control for general exploration (‘Inactive’). CeA ChR2 rats reliably worked to hear their shock-associated CS+ sound repeatedly, making >300% greater nosepokes for CS+ presentation than for their CS− presentation (homecage sound) (Fig. 4b). By contrast, eYFP rats worked at much lower levels, and did not significantly discriminate between CS+ and CS− sounds (1.2:1 ratio).

### Shock-rod attraction activates mesocorticolimbic circuity

We next assessed what brain circuitry was activated in CeA ChR2 rats that were attracted to shock rod, or in ChR2 rats that exclusively pursued either laser-paired sucrose or laser-paired cocaine, by measuring Fos protein expression in mesocorticolimbic brain structures after a final test session.

CeA ChR2 rats pursuing laser-paired sucrose or cocaine showed a pattern of Fos elevation in several limbic structures: ventral tegmental area (>800% activation vs. baseline; Supplementary Table 1), rostromedial NAc shell (>700% activation), and posterior insula (>500% activation). CeA ChR2 rats also showed Fos elevation in dorsolateral neostriatum (>500% activation), and conversely showed an opposite >200% reduction below baseline tissue levels in ventrolateral periaqueductal gray area (PAG) and basolateral amygdala.

**Fig. 5:**
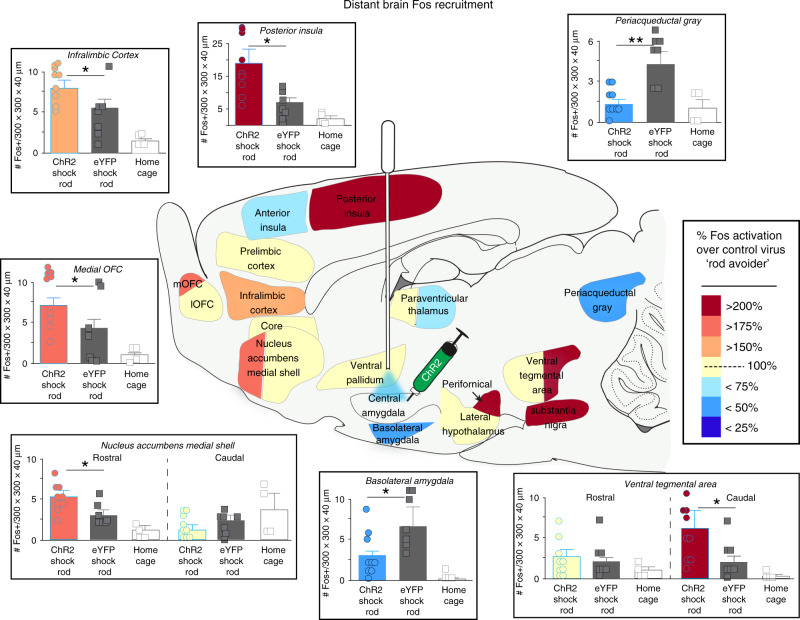
CeA ChR2 shock-rod attraction recruits mesocorticolimbic incentive circuitry. Brain map shows elevated Fos expression in recruited mesocorticolimbic structures in CeA ChR2 rats (*N* = 9, blue outline; colors denote % Fos ChR2 elevation immediately after a final exposure to shock rod compared to eYFP control rats (*N* = 6) and to homecage control baseline rats (*N* = 4). Cortical regions included medial orbitofrontal cortex (mOFC; one-way ANOVA between baseline homecage, eYFP, and ChR2 Fos: *F*_2,33_ = 4.28, *p* = 0.02, Bonferroni-corrected pairwise ChR2 vs. eYFP *t*-test: *p* = 0.025), far-posterior insula (one-way ANOVA: *F*_2,33_ = 4.28, *p* = 0.02, Bonferroni-corrected pairwise ChR2 vs. eYFP *t*-test: *p* = 0.03), and infralimbic cortex (one-way ANOVA: *F*_2,33_ = 4.38, *p* = 0.02, Bonferroni-corrected pairwise ChR2 vs. eYFP *t*-test: *p* = 0.012). Subcortical structures included nucleus accumbens subregions: rostromedial quadrant of medial shell shown (*F*_2,33_ = 4.96, *p* = 0.01, Bonferroni-corrected pairwise ChR2 vs. eYFP *t*-test: *p* = 0.018), caudal half of medial shell (*F*_2,33_ = 0.58, *p* = 0.57), core (NAc core (all subregions combined); *F*_2,33_ = 0.2, *p* = 0.82), ventral pallidum (*F*_2,33_ = 0.57, *p* = 0.57), perifornical area in lateral hypothalamus (*F*_2,33_ = 3.13, *p* = 0.049, Bonferroni-corrected pairwise ChR2 vs. eYFP *t*-test: *p* = 0.014), basolateral amygdala (*F*_2,33_ = 2.63, *p* = 0.04, Bonferroni-corrected pairwise ChR2 vs. eYFP *t*-test: *p* = 0.012), caudal ventral tegmental area (*F*_2,33_ = 4.13, *p* = 0.02, Bonferroni-corrected pairwise ChR2 vs. eYFP *t*-test: *p* = 0.012), substantia nigra pars compacta (*F*_2,33_ = 5.2, *p* = 0.002, Bonferroni-corrected pairwise ChR2 vs. eYFP *t*-test: *p* = 0.02), and periacqueductal gray (*F*_2,33_ = 5.85, *p* = 0.007, Bonferroni-corrected pairwise ChR2 vs. eYFP *t*-test: *p* = 0.004). Also see Supplementary Table 1. Bar graph data shown as mean number and SEM of Fos-expressing neurons in that structure per 300 × 300 × 40 micron (µm) sampling box. *p < 0.05, **p < 0.01.

CeA ChR2 shock-rod attraction induced a similar pattern of mesocorticolimbic activation, with a >400% Fos elevation above eYFP control levels in the midbrain ventral tegmental area (VTA), particularly in the caudal half of VTA (Fig. 5), and >200% elevation in nearby substantia nigra pars compacta (SNc), consistent with activation of dopamine projection neurons^20^. CeA ChR2 shock-rod rats also showed >180% elevation in the rostral medial shell of nucleus accumbens (NAc). NAc Fos was not elevated in the caudal half of medial shell, nor in either rostral or caudal NAc core. In neostriatum, CeA ChR2 Fos was elevated by 200%, particularly in the dorsolateral quadrant of neostriatum. In the basal forebrain, >200% elevation was found in the perifornical region of lateral hypothalamus. In limbic cortex regions, CeA ChR2 Fos was elevated ~175% in medial orbitofrontal cortex (mOFC) and >250% in posterior insula over eYFP levels.

Conversely, shock-rod avoidance among eYFP rats showing defensive behavior was associated with a separate pattern of Fos elevation over CeA ChR2 levels. Specifically, eYFP rats had >400% Fos elevation in the ventrolateral periacqueductal gray (PAG), >240% elevation in the basolateral nucleus of amygdala, and >125% Fos elevation in the bed nucleus stria terminalis (BNST) (Supplementary Table 1).

### CeA stimulation potentiates Pavlovian fear responses

**Fig. 6:**
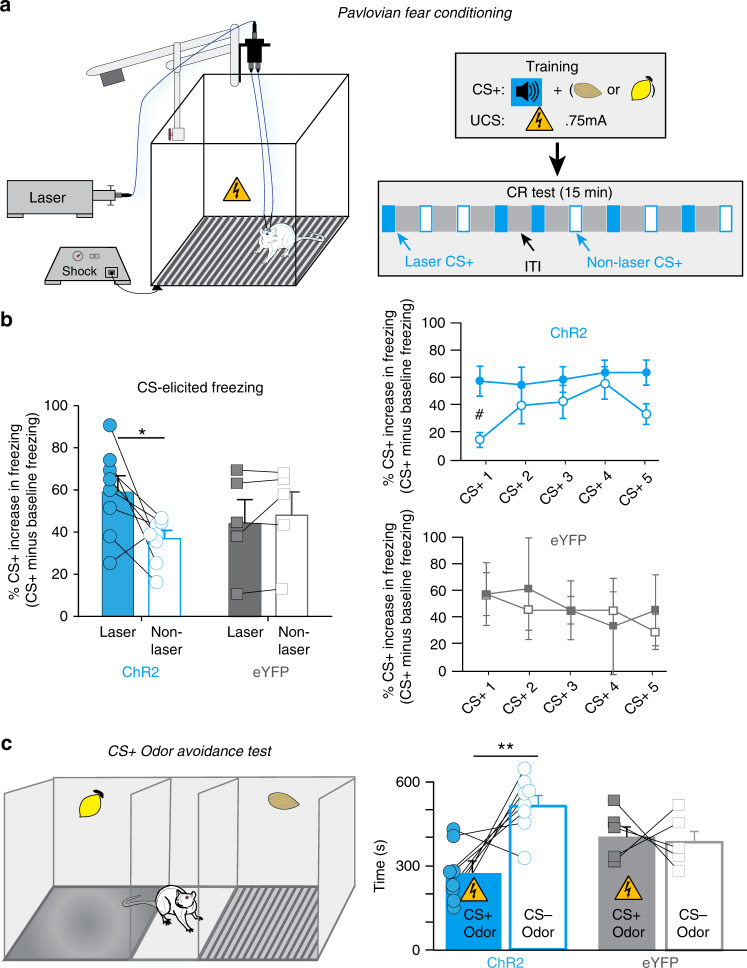
CeA stimulation during Pavlovian fear conditioning. **a** Traditional Pavlovian fear conditioning paradigm: defensive freezing CR elicited by a CS+ tone that predicts unavoidable footshock UCS in Pavlovian chamber. Contextual odor CS+ (almond or lemon scent) was also paired with UCS shock chamber. Right shows training stimuli and session timeline. **b** Freezing conditioned response (CR) during subsequent test session without UCS (% CS-elicited freezing minus % baseline freezing; ChR2 rats, *N* = 8, eYFP rats, *N* = 5, two-way ANOVA, trial type × virus interaction: *F*_1,11_ = 5.07, *p* = 0.036; Bonferroni-corrected pairwise ChR2 laser vs. nonlaser trials *t*-test: *p* = 0.035). Right graphs show individual freezing CRs elicited by CS+ presentation over pre-CS+ baseline (ChR2: two-way repeated measures ANOVA, effect of laser: *F*_1,7_ = 7.36, *p* = 0.03, 1st trial laser vs. nonlaser freezing: Bonferroni-corrected pairwise *t*-test: ^#^*p* = 0.034; eYFP: two-way repeated measures ANOVA, effect of laser: *F*_1,4_ = 0.98, *p* = 0.38). **c** Odor-place avoidance test, with separate compartments containing either CS+ odor (paired with footshock) or CS− odor (paired with homecage). Time spent in either CS+ or CS− side (CeA ChR2 rats, *N* = 8 vs. eYFP rats, *N* = 5, two-way ANOVA, virus × CS+ odor interaction: *F*_1,11_ = 6.06, *p* = 0.03, Bonferroni-corrected pairwise CS+ vs. CS− time, ChR2: *p* = 0.04, 95% CI: −393, −77, *d* = −1.25, eYFP: *p* = 0.2). Data represent mean and SEM. *p < 0.05, **p < 0.01.

CeA has well-known roles in fear learning and defensive motivation, as well as in reward motivation^21–23^, but these are usually tested in different situations. Many fear-related amygdala studies use a Pavlovian conditioned freezing situation, in which an auditory CS+ sound predicts a footshock unconditioned stimulus (UCS) that is uncontrollable, inescapable, and of relatively high magnitude. We therefore assessed if such situations could cause the motivational effects of paired CeA ChR2 stimulation to flip valence from positive to negative in naive CeA ChR2 and eYFP rats, including some that had previous shock-rod experience. During Pavlovian fear training, a 10-s tone (CS+) predicted an unavoidable footshock UCS (0.75 mA, 500 ms) (Fig. 6a*)*. CeA laser illumination began with CS+ onset and continued through UCS footshock (40 Hz, 10 mW). A distinctive olfactory contextual cue (CS+_Context_ scent) was also paired with the Pavlovian fear conditioning chamber.

During test sessions on another day, CS+ tones were presented alone (without footshock UCS), and elicited freezing as Pavlovian conditioned responses (CRs) in both ChR2 and eYFP rats (Fig. 6b*;* ChR2 (*N* = 8) and eYFP (*N* = 5) freezing baselines; two-sided unpaired *t*-test: *t*_11_ = 3.12, *p* = 0.01). CRs were assessed as percent increase in freezing over pre-CS+ baseline levels by the same rat (i.e., normalized to avoid pre-existing differences between groups). Some CS+ presentations were accompanied by CeA laser as during training, whereas other test presentations of CS+ occurred without laser, to assess whether CeA ChR2 laser stimulation altered the expression of freezing CRs elicited by CS+s. Results showed an interaction between CeA laser activation and ChR2/eYFP groups in duration of freezing CRs elicited by CS+s (Fig. 6b), with ChR2 rats emitting longer duration freezing CRs when CS+s were accompanied by CeA laser than when CS+s occurred without laser. This pattern indicates that concurrent CeA ChR2 stimulation magnified the expression of freezing CRs.

Independently, avoidance of the olfactory CS+_Context_ cue associated with Pavlovian footshock UCS was examined in a separate test of place avoidance (Fig. 6c). CeA ChR2 rats displayed avoidance of the place scented with CS+_Context_ odor, whereas eYFP rats did not (eYFP failure to show CS+_Context_ avoidance is consistent with other reports of context-specificity for odor-footshock conditioning, as very different chambers were used here for Pavlovian fear training and odor-place tests^24^). Avoidance of CS+_Context_ by CeA ChR2 rats indicates CeA ChR2 laser stimulation enhanced the acquisition of Pavlovian contextual fear learning during training, as laser was never administered during olfactory avoidance tests and could not have magnified avoidance CR expression. Thus overall, our results suggest CeA ChR2 stimulation in a traditional Pavlovian fear conditioning paradigm can increase both the acquisition and expression of Pavlovian defensive CRs.

### Failure of CeA laser self-stimulation

**Fig. 7:**
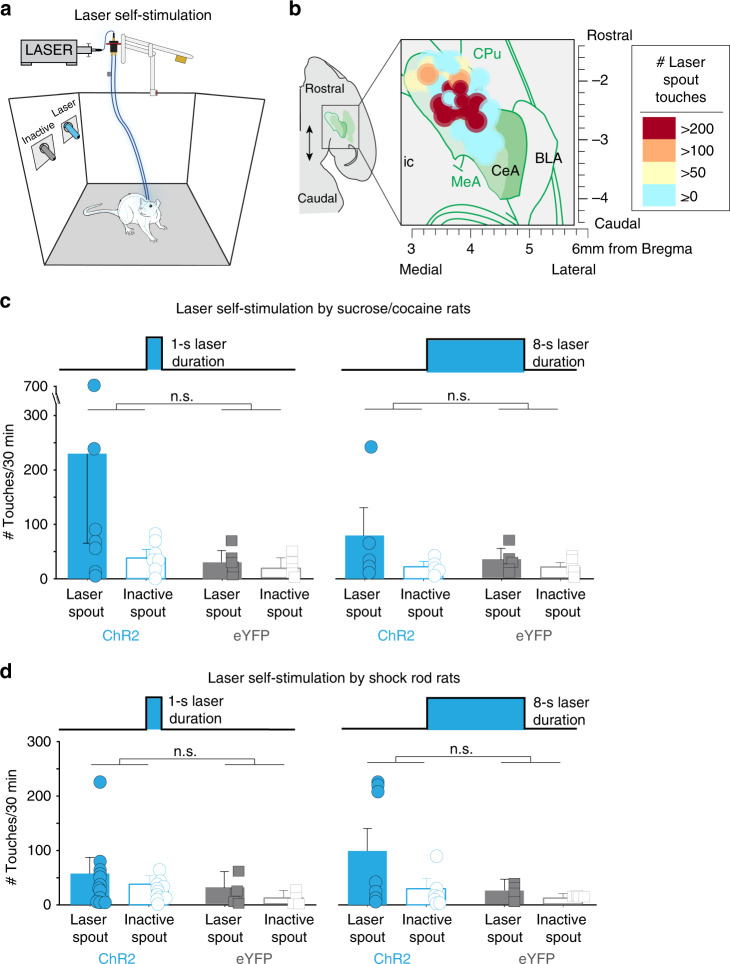
CeA ChR2 stimulation is unreliable as reinforcer alone. **a** Laser self-administration task, in which touching a “laser spout” earned a laser stimulation (1-s or 8-s duration) and touching a separate “Inactive spout” earned nothing. **b** Placement map shows horizontal view of CeA optic fiber placements (size determined be average Fos plume from Fig. 1) with color of each placement indicating an individual rat’s # of laser self-stimulations per 30-min test. **c** Total laser self-stimulations earned by rats from sucrose/cocaine experiments (ChR2 rats, *N* = 10 vs. eYFP rats, *N* = 5, 1 s laser duration, left: two-way ANOVA, virus × laser interaction: *F*_1,15_ = 1.71, *p* = 0.21; 8 s laser duration, right: two-way ANOVA, virus × laser interaction: *F*_1,15_ = 2.06, *p* = 0.18). **d** Total laser self-stimulations earned by rats from shock-rod experiments (ChR2 rats, *N* = 18, eYFP rats, *N* = 6, 1 s laser duration, left: two-way ANOVA, virus × laser interaction: *F*_1,18_ = 0.07, *p* = 0.8; 8 s laser duration, right: two-way ANOVA, virus × laser interaction: *F*_1,18_ = 1.93, *p* = 0.18). All data represent means and SEM; n.s., not significant.

Does CeA ChR2 stimulation have valence or motivational value of its own? We assessed CeA ChR2 valence (alone without shock, sucrose, or cocaine) in laser self-stimulation tests for all rats above, using a spout-touch task. Touching one empty-metal spout earned brief laser illuminations (either 8-s or 1-s duration), whereas touching a different spout earned nothing (and merely served as a baseline measure of exploration) (Fig. 7a)^18^. We found that only a minority of CeA ChR2 rats in the sucrose/cocaine group (3 of 10) and the shock-rod group (4 of 19) met criteria for robust laser self-stimulation (defined as greater than twice as many touches on laser spout as on nonlaser spout, and >50 touches/self-stimulations per session). These seven self-stimulating ChR2 rats earned ~100–300 laser stimulations per session. The remaining 22 ChR2 rats made only 10–40 touches on both spouts, similar to eYFP rats. Thus, overall, the CeA ChR2 rats as a combined group failed to self-stimulate CeA laser significantly (Fig. 7c, d).

This general lack of self-stimulation was notable since laser had powerfully controlled pursuit of shock rod, sucrose or cocaine, even in the same ChR2 rats that failed to self-stimulate. CeA ChR2 rats that did self-stimulate from the shock-rod group failed to show greater shock-rod attraction than non-self-stimulators (*N* = 4 self-stimulators, *N* = 14 non-self-stimulators; two-sided unpaired *t*-test: *t*_16_ = 0.15, *p* = 0.88), and ChR2 self-stimulators from the sucrose/cocaine groups showed no stronger pursuit of their laser-paired sucrose/cocaine reward than other CeA ChR2 rats that failed to self-stimulate (*N* = 7, laser-spout preference × laser-paired sucrose/cocaine preference, Pearson correlation: *r* = −0.17, *p* = 0.69). ChR2 self-stimulators from the Pavlovian fear conditioning group showed laser potentiation of defensive freezing CRs as strong as non-self-stimulators. However, the three strongest laser self-stimulators from shock-rod group chewed more on the shock rod than non-self-stimulators (140 ± 68 s cumulative chewing duration for self-stimulators vs. 31 ± 16 cumulative s for rats that failed to self-stimulate; *t*_16_ = 2.4, *p* = 0.029, 95% CI: −205, −13, *d* = 1.03).

## Discussion

Pairing CeA ChR2 stimulation with sucrose, cocaine, or shock encounters produced strong motivation that switched between positive valence and negative valence, depending on situation. CeA ChR2 stimulation paired with earning sucrose produced single-minded pursuit and consumption focused on sucrose while the rats ignored intravenous cocaine. CeA ChR2 pairing with cocaine for other rats produced pursuit and consumption focused solely on cocaine while they ignored sucrose. CeA ChR2 pairing with shock-rod encounters produced maladaptive attraction to repeatedly approach, touch, and even nibble the shock rod, despite consequently receiving multiple electric shocks.

Our shock-rod findings reveal that a stimulus with aversive properties can become an incentive target when paired with appropriate limbic activation, leading rats to subject themselves repeatedly to noxious shocks in an apparently compulsive fashion. The aversive shock from the rod was itself an important component of CeA ChR2-induced attraction, as a nearly identical laser-paired ‘dummy rod’ without shock failed to become attractive. Thus, CeA ChR2 induction of ‘wanting what hurts’ may provide the strongest proof of principle demonstration available so far that strong mesocorticolimbic ‘wanting’ can be induced in complete absence of ‘liking’.

ChR2 expression via human synapsin (hSyn) promoter indiscriminately infects most CeA neurons, regardless of neurobiological type. Future studies could examine whether CeA neuronal sub-populations (e.g., SOM+, PKC+/−, CRF+, or D1 vs. D2 dopamine receptors, etc.), which have been suggested to play distinct roles in motivated behavior^25–29^ make differential contributions to the CeA ChR2 effects on motivation reported here^20,25,30,31^.

CeA ChR2 stimulations also recruited neurobiological activation among other structures within mesocorticolimbic circuitry to control pursuit of sucrose, cocaine and shock-rod targets. For example, within nucleus accumbens, maladaptive attractions recruited Fos elevation especially in the rostral half of medial shell, which contains a functional hedonic hotspot where opioid, endocannabinoid and related neurochemical signals enhance ‘liking’ reactions, and which is especially implicated in generating positively-valenced motivation even when unaccompanied by ‘liking’^32–34^. In limbic cortex, Fos was also elevated in an anteromedial subregion of orbitofrontal cortex and a posterior subregion of insula; both of those cortical subregions also contain hedonic hotspots^35,36^. This suggests mesocorticolimbic structures traditionally associated with positive valence functions were recruited in order to mediate maladaptive attraction to the shock rod. By contrast, control eYFP rats that fearfully avoided the shock rod, and instead emitted defensive treading, recruited activation of different limbic structures, such as bed nucleus of stria terminalis (BNST), basolateral amygdala, and midbrain periaqueductal gray, which are implicated in anxiety, fear and pain^37–39^.

Several observations suggest that mesolimbic incentive salience, or ‘wanting’, was a psychological contributor to CeA ChR2 attraction. One signature feature of incentive salience attribution is to make Pavlovian cues attractive themselves. Such incentive cues become sought out when absent, and elicit approach when present^40,41^. For example, here CeA ChR2 rats sought out an auditory cue associated with shock rod when absent, showing willingness to work in a new nose-poke task to hear the CS+ sound associated with shock-rod encounters. Similarly, CeA ChR2 rats that were initially unable to see the shock rod due to an occluding barrier, were willing to climb over the barrier to reach the rod.

Consistent with cue attraction, CeA ChR2 rats interacted with the shock rod in ways suggesting the rod had become an attractive cue for shock, rather than seeking out electric shocks per se. That is, ‘wanting what hurts’ may not be the same as ‘wanting to be hurt’. For example, CeA ChR2 rats did not simply throw themselves upon the rod as though seeking shock, but instead continuously examined the rod with close sniffs, bringing face and paws close to the rod. CeA ChR2 rats even sometimes appeared to hold back their paw from the rod for a moment as though avoiding shock, yet eventually their fascination would bring paw or nose too close, and so receive a shock.

Another signature feature of incentive salience is that consummatory behaviors often become directed toward a CS+ cue, such as nibbling on a metal lever or porthole associated with reward^17,42^. Here, CeA ChR2 rats typically nibbled or bit their shock rod, and others nibbled their laser-paired cocaine or sucrose portholes more than their nonlaser reward porthole^9,10^. Increased consummatory biting may be related to other reports of CeA ChR2 induction of biting in mice, such as by activation of CeA projections to the brainstem parvocellular reticular formation^43^. However, biting here did not appear to simply be either a direct aggressive or motor effect, given that CeA ChR2 rats did not typically bite their laser-paired unelectrified dummy rod or wooden block.

A third feature of incentive salience is that ‘wanting’ intensity is often modulated by relevant physiological states and brain states (appetites, stress, intoxication, etc.)^44–47^. Here, CeA ChR2 attraction to the shock rod appeared to be enhanced and maintained by the current brain state. When laser was discontinued, rod attraction faded quickly and was replaced by avoidance and defensive treading. This suggests that, rather than persisting as a permanent learned attraction, simultaneous pairing of amygdala stimulations with shock-rod encounters are needed to continually re-boost the attractiveness of the rod and keep it ‘wanted’.

CeA ChR2 induction of ‘wanting’ does not necessarily imply that it also enhances ‘liking’. To the contrary, a previous study indicated that CeA ChR2 stimulation failed to enhance orofacial ‘liking’ reactions to sucrose taste, despite increasing ‘wanting’ to pursue sucrose^9^. Further, CeA ChR2 stimulation here potentiated negatively-valenced defensive freezing and avoidance responses in the Pavlovian fear conditioning paradigm. Fear CR potentiation is opposite to what would be expected if CeA ChR2 stimulation caused ‘liking’ of paired targets.

A related question is whether CeA ChR2 stimulation reduced the perceived unpleasantness of shock, given that CeA circuitry regulates analgesia and pain reactions (e.g., CeA SOM+ vs. PKC-delta-expressing neurons^48,49^). Here, ChR2 rats that were attracted to the shock rod still reacted with brief flinching reactions to each shock. CeA ChR2 pairings also potentiated defensive conditioned freezing and avoidance responses in the Pavlovian fear conditioning situation, oppositely to what would be expected from analgesia. Still, it is possible that CeA ChR2 stimulation was accompanied by partial oral analgesia for the shock rod in a few individuals: CeA ChR2 rats tended to emit more biting on the shock rod if they also self-stimulated laser, consistent with a potential correlation between self-stimulation and partial analgesia. However, an alternative explanation is equally possible, namely, that CeA ChR2 pairing induced stronger incentive salience in those individuals, both promoting rod biting and enabling enough incentive attribution to the ordinarily-resistant neutral laser spout to motivate self-stimulation, without necessarily being accompanied by analgesia. We conclude that analgesia, while possibly a minor component contributing to prolonged rod biting, was not a primary mechanism of CeA ChR2 attraction.

Why did stimuli such as sucrose, cocaine, shock rod, or footshock all support induction of strong CeA ChR2 motivations, whereas other laser-paired stimuli (e.g., wooden block, dummy rod, or an empty-metal spout for laser self-stimulation) usually evoked weaker or no motivation? One potential explanation is that the eligible target stimuli were all motivationally potent even before laser pairing, in the sense that they could have served as affective unconditioned stimuli (UCSs) to establish motivated Pavlovian conditioned responses. Such affective UCSs could be expected to recruit activation in corresponding mesocorticolimbic circuitry. It is possible that stimulation of CeA ChR2 neurons together with simultaneous UCS-activation of mesocorticolimbic circuitry combines synergistically to create stronger ChR2-induced motivation, which then becomes narrowly focused on the associated target. By contrast, relatively neutral stimuli, such as a self-stimulation spout, dummy rod or wood block, fail to trigger much mesocorticolimbic activity and consequently may remain weaker targets for CeA ChR2-paired motivation.

Our finding that CeA ChR2 pairings potentiated negatively-valenced defensive freezing and avoidance CRs in the traditional Pavlovian fear situation is consistent with many reports of CeA involvement in Pavlovian fear learning^50–52^, but contrasts with CeA ChR2 induction of single-minded appetitive pursuit of cocaine, sucrose, or shock rod here, and other demonstrations of CeA roles in appetitive motivation^53^. CeA ChR2 motivations thus appeared to reverse here between positive and negative valence in different situations, sometimes even within the same individual rat: inducing positive incentive attraction to the shock rod, but amplifying negative fearful reactions to Pavlovian cues for footshock.

What determines switches in CeA valence? One possible explanation is that situational and target stimulus factors interact with CeA neuronal stimulation to determine the valence, as well as intensity, of motivational salience imparted to the paired target. Motivational salience is known to be able take two forms of opposite valence: positively-valenced incentive salience or negatively-valenced fearful salience^45,46^. Incentive salience makes the attributed target more powerfully ‘wanted’ and attention-grabbing, able to elicit approach and trigger seeking and reward consumption. Fearful salience makes its target equally attention-grabbing, but as a potential threat percept that elicits defensive reactions, including the antipredator reaction of defensive treading–burying observed here^54,55^. In humans, mesolimbic fearful salience has also been suggested to contribute to human paranoia^45,56^.

Several features of the Pavlovian fear conditioning situation might have helped tilt the CeA ChR2 valence balance toward fearful salience, whereas the shock-rod situation remained biased toward incentive salience. For example, negative valence in Pavlovian fear conditioning might dominate in part because the noxious footshock was unavoidable and inescapable, and was not spatially localizable to a place within the chamber^57^. By contrast, shocks from the rod were spatially localizable and under voluntary control, occurring only when the rat actively approached and touched the rod^12,13^. Also, Pavlovian footshocks were physically more intense at 0.75 mA than the 0.2–0.5 mA shocks from the shock rod (however, shock-rod shocks were often received on mouth and tongue, which may somewhat offset the higher intensity of footshocks). Future studies will be needed to explore the roles of these or other factors in determining the CeA ChR2 valence.

Do maladaptive attractions described here have potential clinical implications? Important features of addictive motivation include maladaptive motivated pursuit that becomes focused narrowly on the addictive target^58,59^, escalation of consumption, and persistence despite adverse consequences. Narrowly focused pursuit and escalated consumption were seen here and in previous effort-breakpoint studies^9–11^. Our results show that single-minded CeA ChR2 pursuit can be generated and focused narrowly on an incentive target of experimenter’s choice, arbitrarily creating either a ‘sucrose addict’ that ignores cocaine, a ‘cocaine addict’ that ignores sucrose, or even maladaptive attraction to a painful shock rod.

In humans, conceivably, sufficient endogenous CeA activations paired with affective targets might eventually produce similar focused, addictive motivations in susceptible individuals. CeA-induced shock-rod attraction further indicates that an aversive stimulus can itself become a target of compulsive attraction, under certain conditions that recruit mesocorticolimbic incentive circuitry. Maladaptive attraction occurred here without need of hedonic reward, and in absence of any pre-existing habits, in contrast to some contemporary models that posit those features to be required for addictive compulsions^60–62^.

Beyond addictions, CeA ChR2 induction of ‘wanting for what hurts’ may also suggest a potential alternative explanation for some cases of pursuit of pain or harm, such as self-cutting. Although traditional explanations of self-harm typically rely on coping strategies^63,64^, our results suggest that, under some conditions, maladaptive ‘wanting’ can occur directly via incentive motivation processes recruited and focused on a pain-associated target.

Finally, our results showing CeA ChR2 reversal between positive attractions and negative fear potentiation underscores the potential motivational plasticity of CeA circuitry, suggesting the possibility of multiple affective modes for CeA neuronal function. That is, the motivational evaluation of a stimulus may not reside either inherently in the encountered physical stimulus or simply in a momentary CeA activation, but rather in a brain’s individualized representation of their combination, flexibly gated by situational factors.

## Methods

### Animals

Male and female rats (*N* = 55; female Sprague-Dawley = 37; male Sprague-Dawley *N* = 6; female Long Evans Hooded *N* = 12) that weighed between 250 and 300 g before surgery were housed in rooms maintained at ~21 °C on a reverse 12-h light/dark cycle; males and females were housed in separate rooms and always tested separately in clean chambers. Rats had ad libitum access to water and food (Purina Lab Chow) in their home cages throughout the experiment. Prior to experiments, rats were handled at least 5 days for 10 min each day. The University of Michigan’s Committee on the Use and Care of Animals approved all procedures.

### Optogenetic virus infusion and optic fiber implant

Each rat was anesthetized with 5% isoflurane anesthesia, and received atropine (0.04 mg/kg; Henry Schein) prior to surgery, and was maintained at 2–3% isoflurane throughout the surgery. A 0.75 µl volume of optogenetic virus containing a gene for channelrhodopsin with human synapsin promoter (AAV5-hSyn-ChR2-eYFP, *n* = 39) was microinjected bilaterally into the CeA (A/P from Bregma in mm: −2.4, M/L: 4, D/V: −7.6 with mouth bar set to −3.3; 0.1 µl/min for 10 min microinjection). Sites were slightly staggered across individuals to be distributed throughout CeA. Control group rats received an optically inactive virus at similar bilateral sites in CeA (eYFP; AAV5-hSyn-eYFP, *N* = 16). In the same surgery, all rats were implanted with bilateral optic fibers aimed 0.3 mm above the intended CeA site, and fibers were secured to skull screws with a dental acrylic headcap. Rats were subcutaneously injected with cefazolin sodium (60 mg/kg, Henry Schein) to prevent infection, and carprofen (5 mg/kg, Henry Schein) as a post-surgical analgesic.

### Intrajugular catheter implantation

In a separate surgery 2 weeks later, rats intended for cocaine self-administration tests (*N* = 19; females = 14; males = 5) were anesthesized again as above and were implanted with an intravenous catheter in the jugular vein^10^. Silastic intrajugular catheters (0.28 mm internal diameter) were threaded into the right jugular vein, then passed subcutaneously along the dorsal neck and secured to an anchor exiting from the dorsal mid-scapular region. Rats were allowed 10 days recovery before beginning any behavioral tests. Intrajugular catheters were flushed daily with 0.2 ml sterile isotonic saline solution containing 5 mg/ml gentamicin sulfate (Sparhawk, KS) for 2 weeks, and by sterile saline alone thereafter, to prevent infections or clogs. Catheter patency was tested once before behavioral testing, and again after the end of all tests, by intravenous injection of 0.2 ml methohexital sodium to induce ataxia (20 mg/ml in sterile water, JHP, MI). Rats that became ataxic within 10 s were considered to have a patent catheter and included in analyses.

### Sucrose vs. cocaine instrumental choice

Choice training and tests (sucrose vs. cocaine) were carried out in modified MedAssociates chambers (30.5 × 24.1 × 21.0 cm) with clear Plexiglas floors, which contained two instrumental nosepoke portholes on a side wall. Nose-poking into either porthole was detected by infrared beams and recorded by MedPC software. For some rats, these portholes were retractable, so that they were usually flush with the wall and occluded, but the outer rim could enter through the wall to protrude in the chamber, while the inner center did not protrude, to present an active porthole able to earn cocaine or sucrose. After the presentation, the porthole was retracted again to disappear, while the other porthole entered the chamber or a time-out ensued. This was meant to mimic the presentation and disappearance of retractable levers in previous studies that used levers as instrumental manipulanda^10,14^. For other rats, two standard portholes remained fixed in place throughout the entire session, to mimic other previous studies in which nosepokes earned intravenous cocaine^10,60^. This difference ensured that our results were not limited to either procedure. An infusion pump outside the chamber delivered intravenous liquid cocaine delivery via tygon tubing. Nosepokes into the cocaine porthole always earned a 50 µL intravenous infusion of 0.3 mg cocaine (weight of the salt; donated by NIDA, Lot# 13722-21C) per kg weight of the rat, dissolved in isotonic saline, infused over a 2.8-s period. Sucrose pellets were delivered into a recessed dish in the chamber wall between the portholes via food hopper. Since choice between cocaine and sucrose may partly depend on the palatability of the sucrose pellet^15,65^, we used two different sucrose pellets for different rats. For some rats, nosepokes on their sucrose porthole earned a 45 mg nearly pure-sucrose pellet (*N* = 8; LabTabs^TM^, TestDiet, Richmond, IN), and for other rats, it earned an even more preferred 45 mg sucrose candy pellet that also contained milk fat and casein as well as sucrose (*N* = 8; AIN-76A, TestDiet, Richmond, IN). A video camera placed below the transparent floor recorded all behavior for subsequent off-line analysis of consummatory behaviors, such as chewing on the portholes^10^.

Rats were first trained in 60-min daily sessions with a single active porthole and single daily reward for 6–10 days until each rat attained a criterion of earning a cumulative total of 50 sucrose rewards and 50 cocaine rewards. Training days alternated between earning either sucrose exclusively or cocaine exclusively, each through nosepokes on its own instrumental porthole, until criterion was reached for both outcomes. Subsequent tests used simultaneous 2-choice presentations of both portholes, allowing either or both rewards to be earned. Some rats were randomly chosen and designated to be permanently ‘Laser+Cocaine rats’. Others were permanently designated to be ‘Laser+Sucrose rats’. All rats had met criterion already for earning both rewards. For each rat, bilateral blue laser illumination (473 nm, 10 mW, 25 Hz, 8-s duration) for optogenetic CeA excitation was always paired with earning their laser-designated reward, beginning immediately with nosepoke registration and continuing as they received the cocaine infusion or consumed the sucrose pellet (Laser + Sucrose: *N* = 5 ChR2 and *N* = 2 control eYFP; Laser + Cocaine: *N* = 6 ChR2 and *N* = 3 control eYFP). Earning their alternative reward was never accompanied by CeA laser.

On given training days 1–8, a rat could earn only cocaine, or else only sucrose, by nosepokes into its particular porthole designated for that reward^14^. The other porthole produced no outcome during that day (if porthole was fixed) or was not present (if retractable). The next day, nosepokes into the second porthole earned the alternative reward, while the first porthole was inactive or not present. This pattern continued until the end of training (when rats earned a total of 50 cocaine and 50 sucrose rewards). Some rats began training with cocaine reward on the first day, while other rats began with their sucrose reward. All rats also received auditory Pavlovian CS cues via wall speaker to mark successful earning of each porthole’s reward outcome (tone or white noise; 8 s); assignment of tone/white noise auditory cues to sucrose or cocaine was always consistent for a given rat, but balanced across rats. Amount of days to reach criterion of earning 50 rewards was equal whether laser was paired with sucrose or cocaine (ChR2, *N* = 11 vs. eYFP, *N* = 5; two-way ANOVA: *F*_1,12_ = 1.49, *p* = 0.25).

At the beginning of a 2-choice test session, one randomly selected porthole was first presented or made operative alone until its reward was earned. Then after a 20-s time-out the alternative porthole was presented until the rat earned its other reward. This sequence was repeated again, so the rat earned two cocaine rewards and two sucrose rewards (forced-sampling) immediately prior to making a choice. This was done to be sure that each rat re-experienced both rewards, ensuring equal priming, and to avoid danger of the rat becoming trapped into simply choosing the first reward encountered.

Subsequently during each of the 2-h session, both portholes were always presented simultaneously, allowing a 2-choice decision, so the rat could choose which reward it preferred to earn. These simultaneous presentations were repeated for up to 10 times each session, allowing 10 consecutive choices to be made. Once a choice was made and earned by a nosepoke, its outcome was delivered (sucrose pellet or 0.3 mg/kg cocaine infusion as in training) accompanied by its associated auditory cue. After each choice was made, a 10-min time-out was imposed before the two portholes again became operative or presented^14^. Each rat also received bilateral CeA laser with each of its individually designated outcome, either sucrose or cocaine, but never with the alternative outcome (473 nm, 10 mW, 25 Hz, 8 s bin illumination). This entire choice procedure was repeated daily for another 3 days^66^.

### CeA laser self-stimulation tests

To assess whether CeA ChR2 excitation was an independent incentive or reinforcer by itself, rats were tested for CeA laser self-stimulation. In an active spout-touch task, rats could earn laser illumination on a FR1 basis by actively touching a designated empty-metal spout. Rats were placed in MedAssociates operant chambers in which two novel and empty sipper spouts protruded ~5 inches apart from the back wall of the chamber. Each touch upon a spout closed a circuit between spout and metal grid floor, and was recorded. One spout (designated as ‘laser spout’; spout assignment counterbalanced across rats) delivered a 1 or 8 s CeA laser stimulation each time it was touched (25 or 40 Hz, 10 mW, 1-s duration: *N* = 11, or 8-s duration: *N* = 7). The 1-s pulse duration was used because it has supported robust optogenetic self-stimulation in previous studies^18^. The 8-s pulse duration was assessed in separate tests because it replicated the laser parameters that controlled motivation for laser-paired cocaine or sucrose in 2-choice task above. The second spout never produced laser, and simply served as a control to assess baseline exploratory touches on a similar object. Each session lasted 30 min. Rats were considered to be laser self-stimulators if the made at least twice as many laser spout touches than inactive spout touches, and made >50 touches.

### Laser-paired aversive shock rod

In a separate experiment with different rats (ChR2: *N* = 25; eYFP: *N* = 11), we paired voluntary encounters with an aversive “shock rod” with CeA ChR2 stimulation in order to compare effects of CeA stimulation with a negative-valenced outcome. In this situation, all encounters with shock are under the rat’s instrumental control, and it can conversely choose to avoid shocks. In that sense, instrumental shock pursuit would be similar to instrumental pursuit of sucrose or cocaine rewards, but with an outcome of opposite affective valence (aversive electric shock). The shock rod (1.5 × 1.5 × 9 cm core, wrapped with electrified wire along its full length) protruded 9 cm into one side of a Plexiglas chamber containing 2-cm depth of corn cob bedding scattered on the floor (chamber: 38-cm width × 38-cm length × 48-cm height; bedding: Bed’O’Cobs, Andersons Inc., Maumee). The bedding was present to allow defensive burying behavior, which is normally elicited from rats that encounter the shock rod^12,13,54^. Touching the rod delivered a 0.2–0.5 mA (depending on <0.25- to >1-s duration of contact; measured using in-house ammeter), which continued as long as contact was maintained (duration <1/5th s). Touching the rod was never forced, but each rat touched at least once while exploring the chamber. Any subsequent touches were purely voluntary, as the rod occupied under 2% of the floor area of the chamber. A video camera recorded behavior throughout each session for subsequent off-line analysis.

On the initial shock-rod day, rats were attached to bilateral optic fiber delivery cables, placed into the middle of the chamber and allowed to freely move around and explore the chamber in a 20-min session. Upon first contact with the shock rod, usually with forepaw or sometimes with snout, a mild shock (0.20 mA) was delivered to the skin. The rat typically withdrew contact reflexively and terminated the shock within 50–100 ms. Laser illumination began when any part of a rat approached within 2 cm of the rod (473 nm, 10 mW, 40 Hz (5-ms ON, 20-ms OFF, triggered via MATLAB program), and continued until the rat withdrew further than 2 cm away from the rod. Approaches within 2 cm were 95% of the time accompanied by shock, so laser activation bracketed the shock before and after for a second or so, typically with a 3–8 s total duration. Sometimes the rat touched the rod again before withdrawing, which accounted for the longest laser illuminations. Our intention in this was to paste CeA ChR2 excitation on the entire perceptual encounter with the shock-delivering rod, rather than on the brief (typically < 0.1–0.25 s) shock alone, similar to laser duration in sucrose and cocaine encounters in the previous study. A subset of rats (ChR2: *N* = 8, eYFP: *N* = 6) also heard an auditory Pavlovian CS+ whenever within 2 cm of shock rod and laser was illuminated, with the same duration as laser (tone or white noise, counterbalanced between rats). This was intended to provide an additional sensory CS+ label for encounters with the shock rod. The alternative CS− sound (either white noise or tone) was presented later that day in a separate session the same number of times as the CS-, in a similar chamber with bedding but in a different room and with no rod present. This roughly equated the number of presentations of CS+ and CS− sounds. All behavior was video-recorded for off-line scoring later. Identical shock rod and laser sessions were repeated on days 2 and 3. On the fourth day of training, a laser-extinction session was run, similar to previous days and with the rod still electrified, but no laser illumination was delivered. This laser-extinction test assessed whether CeA ChR2 established learned changes in behavior toward the rod that were enduring, or instead depended on actual CeA ChR2 excitation during the test.

### Instrumental conditioned reinforcement test

The hypothesis that CeA ChR2 promotes motivation in part by attributing incentive salience to cues for the paired UCS target implies that Pavlovian CS+s for an attractive target become attractive themselves. Attraction to the shock rod provides a powerful test of this hypothesis, as it implies that an auditory CS+ label for shock might become paradoxically attractive to CeA ChR2 rats. We assessed the attractiveness of the auditory CS+ associated with shock in CeA ChR2 rats and control eYFP rats by asking if they would learn to perform an instrumental nosepoke response to earn presentations of either the auditory CS+ alone or the equally familiar CS− (ChR2: *N* = 8, eYFP: *N* = 6). This instrumental conditioned reinforcement test occurred on 2 separate days in a MedAssociates chamber. Rats were presented with two novel fixed portholes (these rats had never previously learned to nosepoke for any reward, so porthole nose-poking was an entirely new instrumental response for them). On the CS+ day (balanced order), a nosepoke into one designated porthole earned a 4-s presentation of the auditory CS+ that previously had been paired with shock-rod encounters (FR1; either tone or white noise for different rats; responses were considered ‘CS+ pokes’). Nosepokes into the other porthole produced nothing, and were recorded to assess baseline pokes due to general activity or exploration (‘CS+ Inactive poke’). On the other CS− day, a nosepoke into the active port now produced a 4-s presentation of the CS− sound (white noise or tone; ‘CS− pokes’), while the other port still delivered nothing (‘CS− Inactive poke’). The number of nosepokes in each porthole was recorded. Each daily session lasted 30 min, and order of CS+ and CS− days was counterbalanced.

### Motivated rod approach? Overcoming sudden barrier

To further test whether shock-rod approach by CeA ChR2 rats was flexibly motivated, in the sense of being willing to overcome a novel barrier suddenly placed in their way in order to get to the rod, CeA ChR2 rats and control eYFP rats with 3 days of previous shock-rod experience were given a barrier test. The sessions began with 5 min of free access to the rod as in days 1–3, with both shock and laser conditions activated (473 nm, 10 mW, 40 Hz laser). After 5 min, an opaque barrier (37-cm length × 13-cm width × 13-cm height, cardboard box wrapped in a blue pad) was inserted in the middle of the chamber between the rat and the shock rod, gently nudging the rat if needed to block its access to the rod. The barrier occluded the rat’s view of the shock rod, and physically prevented approach unless the rat actively climbed 13 cm over the barrier to reach the rod. Behavior was video-recorded for another 10 min, for subsequent off-line analysis of climbs, latency to reach the rod, and touches (Noldus Observer XT 12).

### Pavlovian fear conditioning

To further examine CeA ChR2 in a standard defensive fear conditioning paradigm, in which CeA has been implicated in threat learning, naive rats (ChR2: *N* = 8; eYFP: *N* = 5) were trained for 3 consecutive days to learn a Pavlovian association between an auditory CS+ and an unavoidable UCS 0.5-s footshock (0.75 mA)^52^. During training on the first day, after a 3-min habituation period, three CS+/UCS pairings were presented and separated by 60 s fixed inter-trial intervals. The auditory CS+ was a 10-s tone (80 db at 5 kHz), and accompanied by bilateral CeA laser illumination (473 nm, 10 s, 40 Hz, 10 mW) during training. The UCS was 0.5-s footshock scrambled across the grid floor (500 ms, 0.75 mA) that followed immediately after termination of CS+ (did not overlap). In addition, another contextual olfactory CS+ cue was present during shock conditioning trials (either almond or lemon essence, counterbalanced; applied by task wipes (KimTech Science)). The alternative contextual CS− odor was separately presented in the homecage in sessions equal in number and duration. After the three pairings of CS+ on day 1, an additional two pairings were presented on day 2, and a final one pairing was presented on day 3. Pavlovian freezing as a conditioned response (CR) to the auditory CS+ was tested on day 4 in a distinctly different chamber, which had a plexiglass floor (not metal grid) with a different odor (Versaclean) and house light. After a 1-min baseline period, a series of 10 CS+ tones were presented, each separated by 60 s. During five of these presentations (order randomized), bilateral CeA laser illumination was delivered for 10 s concurrently with the CS+ tone (10 s, 40 Hz, 10 mW). The other five CS+ presentations occurred alone, without CeA laser. On a subsequent day, rats were tested for contextual CS+ odor avoidance in a place preference/avoidance chamber for 30 min: one chamber was scented with the footshock-associated contextual CS+ odor, and the other chamber with CS− odor (scented wipes placed underneath the chambers; sides of CS+ odor assignment counterbalanced between rats). The two chambers were also distinguished by different visual patterns on the walls to aid discrimination (stripes or polka dots). Time spent in each compartment was video-recorded and subsequently scored offline using Noldus Observer Software.

### Wooden block/food intake: CeA stimulation and general motivation to eat

We explored the effect of CeA laser stimulation on voluntary food consumption in a 60 min free-intake test. Rats (*N* = 8 ChR2, *N* = 4 eYFP) were tested in a familiar homecage environment with bedding on the floor, and had continuous access to pre-weighed quantities of food (Purina Lab Chow; ~20 g) and water. Behavior was video-recorded, and at the end of each session, remaining chow weight and water volume was recorded again to calculate the amount consumed. A pre-weighed wooden block (~18 g) was also available to allow non-ingestive chewing, and was re-weighed at the end. The first day was considered a familiarization procedure to encourage a reliable baseline. Intake tests were repeated the next 2 consecutive days to obtain baseline vs. laser measures. Laser stimulation was administered only on 1 day, occurring either on day 2 or 3 (ABA or AAB design, counterbalanced across rats) in 15-s ON–9-s OFF alternations (40 Hz; 20-ms ON, 5-ms OFF; 10 mW), and the other 2 days served as baseline comparisons. Cumulative time spent eating, drinking, or chewing during laser and nonlaser sessions was scored offline using Noldus Observer software.

### Histological analyses of virus expression and Fos plumes

Beginning 75 min prior to euthanasia and perfusion, CeA laser stimulations with parameters similar to those that had produced incentive effects were given to rats in sucrose–cocaine choice (*N* = 7) and in shock-rod encounter (*N* = 16) groups. Laser stimulation was either accompanied by cocaine-sucrose choice (ChR2: *N* = 4) or shock-rod situations (ChR2: *N* = 12 and eYFP: *N* = 4) to re-activate CeA-induced systems and behavioral incentive effects simultaneously. Another control group of unoperated and naive rats, never before exposed to any experimental situation, were taken directly from homecage for euthanasia and perfusion, to allow comparison to measure normal baseline levels of Fos expression (in the absence of cocaine, sucrose, shock-rod or related stimuli, and without surgical penetration, gliosis, virus infection or light/heat insults to neural tissue; *N* = 4).

After 75 min from the onset of any above condition, rats were deeply anesthetized with an overdose of sodium pentobarbital (150–200 mg/kg) and transcardially perfused using ice-cold PBS followed by ice-cold 4% PFA. Brains were post-fixed for 24 h in 4% PFA, cryoprotected in 30% sucrose PBS, and coronally sectioned at 40 µm using a cryostat (Leica). For immunohistochemistry, sections were first blocked in 5% normal donkey serum/2% triton-X PBS solution for 30 min, incubated for 24 h in a polyclonal rabbit anti-cfos IgG primary antibody (1:1000, Santa Cruz Biotechnology), followed by 2 h in AlexaFluor anti-rabbit IgG secondary antibody (3:1000, Life Technologies). All sections were mounted, air-dried, and cover-slipped with anti-fade Pro-long gold (Invitrogen). For each CeA placement, images surrounding the fiber optic tip were taken at ×10 magnification, using a Leica microscope and Oasis surveyor software. Immunoreactivity for Fos protein and virus expression were visualized using filters with excitation bands 515–545 and 490–510, respectively. Number of Fos+ (or eYFP+) cells were counted in 15 successive blocks (50 × 50 μm) along eight radial arms that emanated from the fiber optic tip. Counting continued along each arm until at least two consecutive boxes were zero, at which point marked the radius of that arm. Fos elevation was calculated as % change from either of two baselines: (1) Illuminated inactive-virus control levels: equivalent block locations from CeA of eYFP control rats that received laser illumination prior to perfusion similarly to ChR2 rats, or (2) Normal tissue baseline: counts of Fos from CeA in unoperated control brains of normal rats. Fos elevations in ChR2 blocks were denoted in increments of >200% elevation or higher >300% elevation above the respective two mean baselines^10,66^.

### Fos quantification in distributed brain circuitry

Oasis Surveyor software was used to capture tiled images of whole brain coronal section at ×10 magnification pre-determined by Paxinos and Watson brain atlas^67^ and using a filter with 515–545 excitation band to visualize Fos expression. Whole brain images were used to count Fos protein at multiple sites in orbitofrontal cortex, insula, basolateral amygdala, nucleus accumbens core and shell, ventral pallidum, ventral tegmentum, periacqueductal gray, and lateral hypothalamus. For each brain region, three sites each in anterior, posterior, and middle regions were separately counted under treatment-blind conditions. For each site (at each anterior-posterior site), three 100 × 100 × 40 μm boxes were placed onto the coronal brain image in Adobe Photoshop software by those blind to experimental conditions. To ensure site placements were consistent between rats, placement of the three boxes for each subregion were guided by a template plotted on a brain atlas page corresponding to the structure^66^.

### Statistical analysis

Mixed ANOVAs were used to analyze within-group effects (e.g., laser pairings and on/off conditions) and between-group differences (e.g., CeA ChR2 vs. CeA eYFP groups). Significant ANOVAs were followed by parametric paired *t*-tests and independent *t*-tests to analyze specific post hoc comparisons of conditions (using either Bonferroni or Dunnett’s two-sided tests). Data found to not have normal distributions were analyzed with nonparametric one-way ANOVAs followed by nonparametric paired *t*-tests. Each test used a confidence interval of 95% with a significance level of *p* < 0.05, two-tailed. Finally, Cohen’s *d* was used to calculate effect sizes among pairwise comparisons.

### Reporting summary

Further information on research design is available in the Nature Research Reporting Summary linked to this article.

## Supplementary information

Supplementary Information

Peer Review File

Supplementary Movie 1

Supplementary Movie 2

Reporting Summary

## Data Availability

The data that support the findings of this study are made available through NIH figshare public repository, 10.35092/yhjc.c.4939542^66^. Data underlying Figs. 1–7 and Supplementary Figs. 1–5 are also provided as a Source Data file.

## Source data

Source Data File
