# Supplementary material for: The central amygdala recruits mesocorticolimbic circuitry for pursuit of reward or pain
Source: Nat Commun. 2020 Jun 1;11:2716. doi: 10.1038/s41467-020-16407-1 (PMC7264246; doi:10.1038/s41467-020-16407-1)
Supplement: Supplementary file 1 — Supplementary Information [file 41467_2020_16407_MOESM1_ESM.pdf]

Supplementary Information

The central amygdala recruits mesocorticolimbic circuitry for pursuit of reward or pain

Warlow et al.

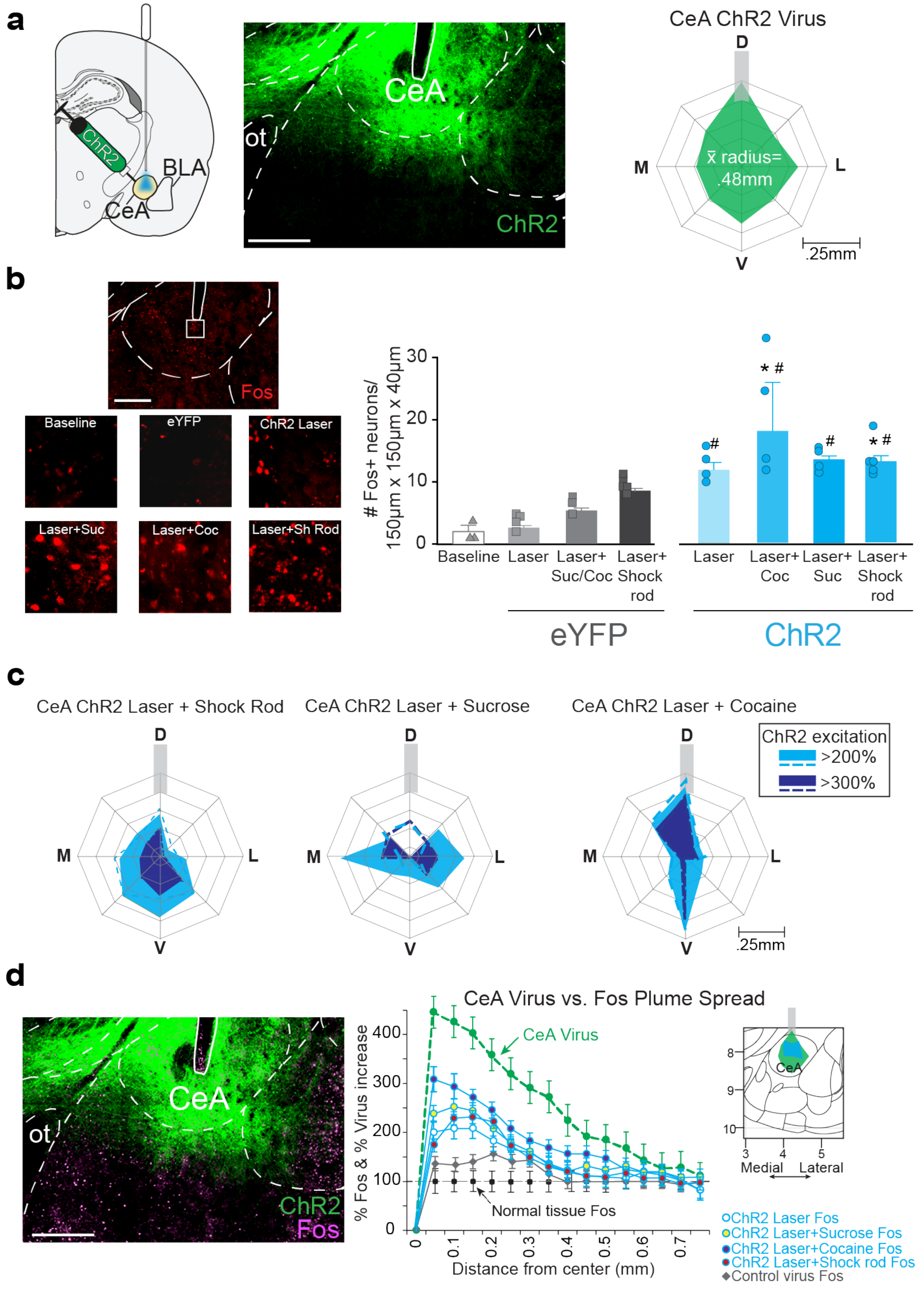

Supplementary Figure 1 CeA ChR2 Virus and Fos Plumes (a) Photomicrograph taken at 10x magnification depicts representative channelrhodopsin (ChR2) virus (AAV5-hSyn-ChR2-eYFP, green, *N=39* rats) within central amygdala (CeA); ot=optic tract; scale bar: 0.5 mm. Right shows average ChR2

virus spread away from fiber optic tip. **(b)** Photomicrographs taken at 10x magnification (scale bar, 0.3 mm) depict Fos protein expression in representative sections (within 150 μm x 150 μm around the optic fiber tip) from either baseline rats (*N=3*), eYFP controls (*N=10*), ChR2 rats receiving non-contingent laser (*N=4*), ChR2 Laser + Sucrose (*N=4*), ChR2 Laser +Cocaine (*N=4*), or ChR2 Laser+Shock rod ("Sh rod", *N=5*). (Sucrose/Cocaine conditions, ChR2 (*N=8*) vs. eYFP (*N=3*), One-way ANOVA: $F_{5,16}$=9.78, p=.000; Shock rod conditions, ChR2 vs. eYFP (*N=5*), One-way ANOVA: $F_{2,16}$=13.2, p=.000). Bonferroni-corrected post hoc comparisons with baseline tissue: ChR2 laser+sucrose, #p=.02, Cohen's *d*=5.1; ChR2 laser+cocaine, # p=.05, Cohen's *d*=1.1; and ChR2 laser+shock rod, # p=.003, Cohen's *d*=8.3. Bonferroni-corrected post hoc comparisons with eYFP rats in same condition: ChR2 laser+sucrose, p= .09, Cohen's *d*=3.0; ChR2 laser+cocaine, *p=.04, Cohen's *d*=2.8; and shock rod ChR2 laser+shock rod, *p=.02, *d*=21. **(c)** The size (radius) of Fos plumes induced by CeA ChR2 Laser, showing functional activation spread; D=dorsal, M=medial, L=lateral, V=ventral). **(d)** Photomicrograph taken at 10x magnification depicts representative ChR2 virus expression and Fos protein expression (scale bar: 0.5 mm). Right graph shows quantitative elevation in virus and Fos as function of distance from fiber tip (CeA Virus: *N=11*, ChR2 Laser Fos: *N=4*, ChR2 Laser+Sucrose Fos: *N=4*, ChR2 Laser+Cocaine Fos: *N=4*, ChR2 Laser + Shock rod Fos: *N=11*, Ctrl eYFP Fos: *N=4*). All data represent mean and standard error (SEM).

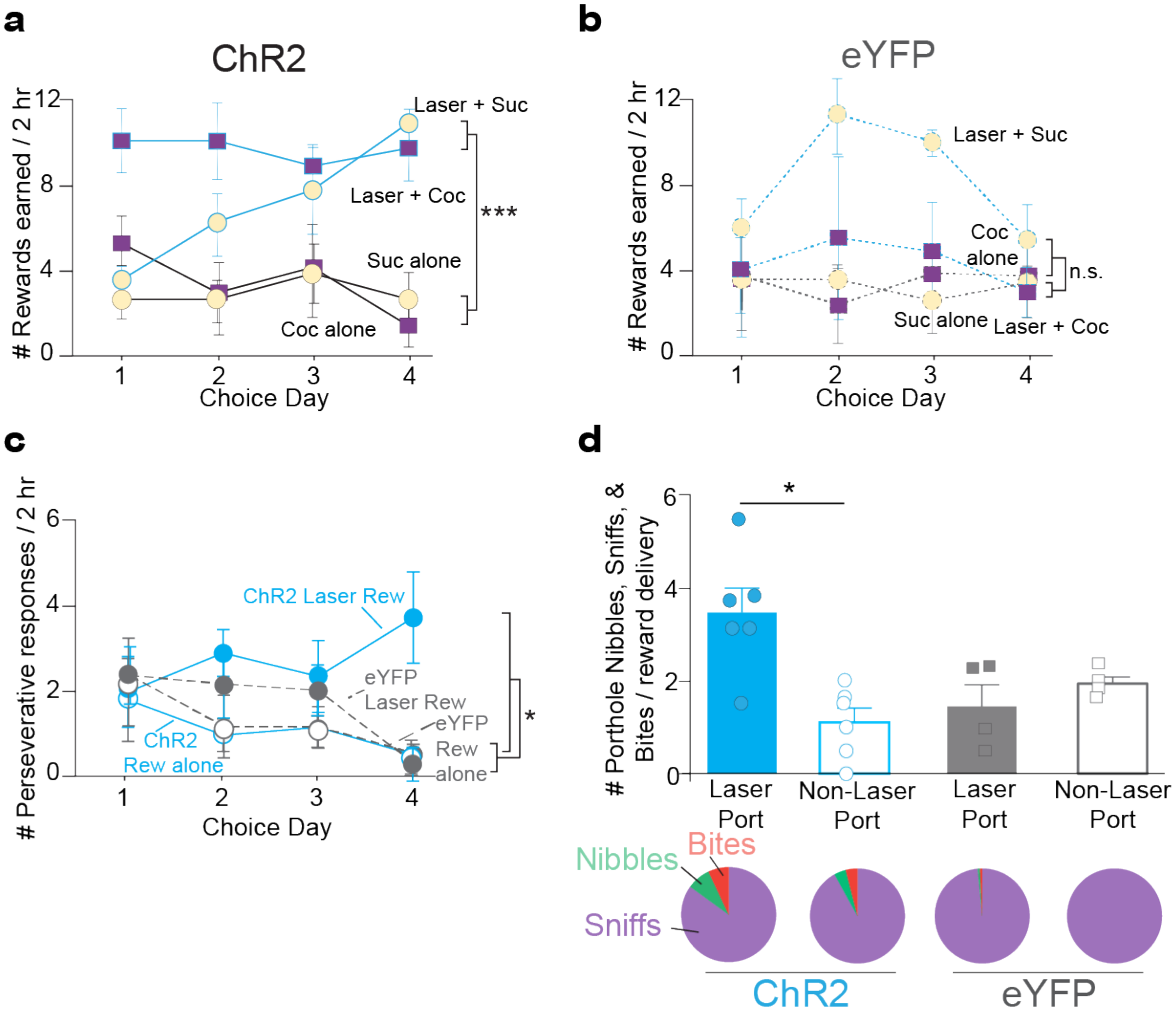

Supplementary Figure 2 CeA ChR2 laser pairing controls pursuit of cocaine versus sucrose **(a)** Earned rewards by CeA ChR2 rats during each sucrose vs. cocaine choice session, Laser+Sucrose (*N=5),* Laser+Cocaine (*N=6*), 2-way repeated measures ANOVA, main effect of laser: $F_{1,7}$=61.3, p=.000. **(b)** Earned rewards by eYFP rats during each sucrose vs. cocaine choice session, Laser+Sucrose (*N=2),* Laser+Cocaine (*N=3*), 2-way repeated measures ANOVA, main effect of laser: 2-way repeated measures ANOVA, $F_{1,3}$=1.35, p=.33, n.s., non-significance. **(c)** Instrumental nose pokes during time-out period ('Perseverate Responses'), ChR2 rats (*N=11*), eYFP rats (*N*=5), 2-way repeated measures ANOVA, laser x virus interaction, $F_{1,10}$=5.1, p=.04. **(d)** Consummatory nibbles, sniffs, and bites directed at portholes during each reward delivery, ChR2 rats (*N= 6)*, eYFP rats (*N=4)*, 2-way repeated measures ANOVA, $F_{1,8}$=9.1, p=.017; Bonferroni-corrected pairwise t-test, laser vs non laser port hole behaviors, ChR2: p=.03, *d*=1.57; eYFP: p=0.8). Pie charts below show proportion of nibbles (green), sniffs (purple), and bites (red) displayed towards each porthole. Data represents mean and SEM. *p<.05 **p<.01 ***p<.001

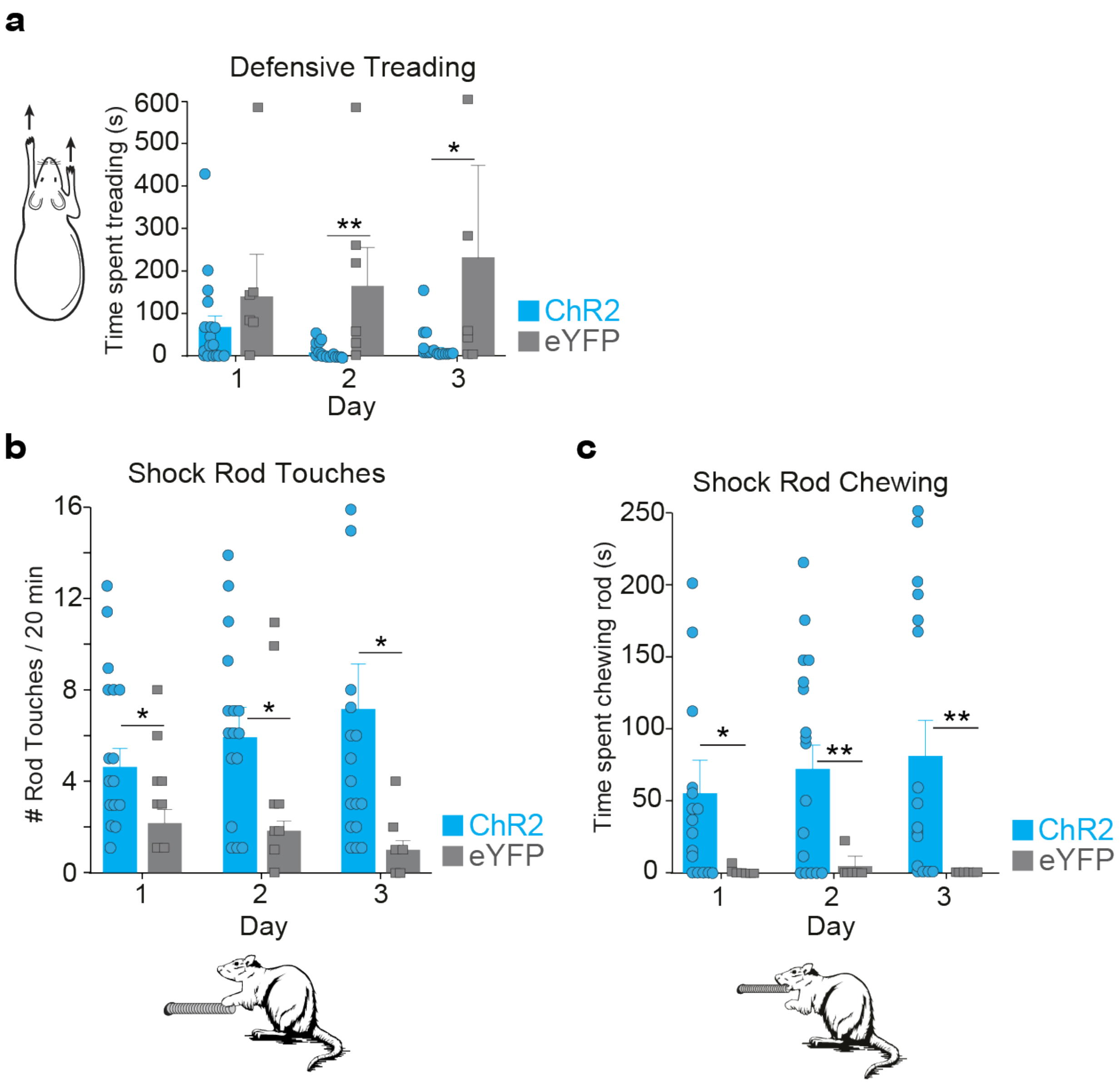

Supplementary Figure 3 All test days of CeA pairing with aversive shock rod. (a) Time spent defensive treading towards an electrified shock rod across 3 test days, ChR2 rats (*N=16*), eYFP rats (*N=6*), 2-way repeated measures ANOVA, main effect of virus: $F_{1,18}$=3.36, p=.04; Bonferroni-corrected two-sided unpaired t-tests, ChR2 vs. eYFP: day 2, p=.02, *d*=0.8; day 3, p=.04, *d*=0.72. **(b)** Shock rod touches across 3 test days, ChR2 rats (*N=16*), eYFP rats (*N=6*) (2-way repeated measures ANOVA, main effect of virus: $F_{1,18}$=4.7, p=.04; Bonferroni-corrected two-sided unpaired t-tests, ChR2 vs. eYFP: day 1, p=.048, *d*=1.2; day 2, p=.003, *d*=1.12; day 3, p=.006, *d*=-1.07). **(c)** Time spent chewing shock rod across 3 test days, ChR2 rats (*N=16*), eYFP rats (*N=6*), 2-way repeated measures ANOVA, main effect of virus: $F_{1,18}$=4.8, p=.04), Bonferroni-corrected two-sided unpaired t-tests, ChR2 vs. eYFP: day 1, p=.04, *d*=0.8; day 2, p=0.001, *d*=1.37; day 3, p=.008, *d*=1.06). Data represents mean and SEM. *p<.05 **p<.01

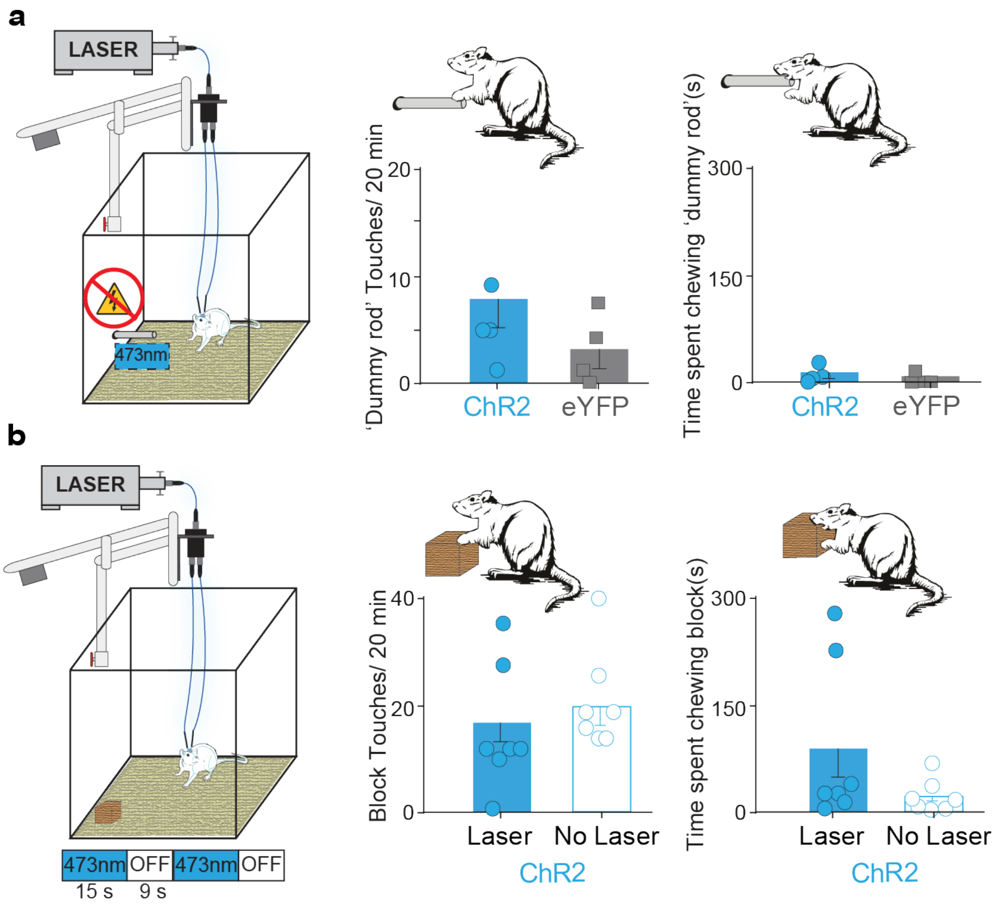

Supplementary Figure 4 CeA ChR2 stimulation does not enhance attraction to neutral objects (a) Apparatus containing a unelectrified "dummy" rod which lacked electrical wiring and did not deliver shock when touched, but was paired with CeA laser stimulation. ChR2 (*N=4*), eYFP (*N=4*); Number of 'dummy' rod touches: two-sided unpaired t-test: t_6=1.47, p=.18; Time spent chewing the 'dummy rod': two-sided unpaired t-test: t_6=1.14, p=.30. **(b)** A separate apparatus containing inedible wooden blocks and cyclic delivery of laser stimulation, ChR2 (*N=7)*, Wooden block touches, laser vs. no laser session: two-sided paired t-test, t_6=1.01, p=.35; Time spent chewing wooden block, laser vs. no laser session: two-sided paired t-test, t_6=0.53, p=.61. Data represents mean and SEM.

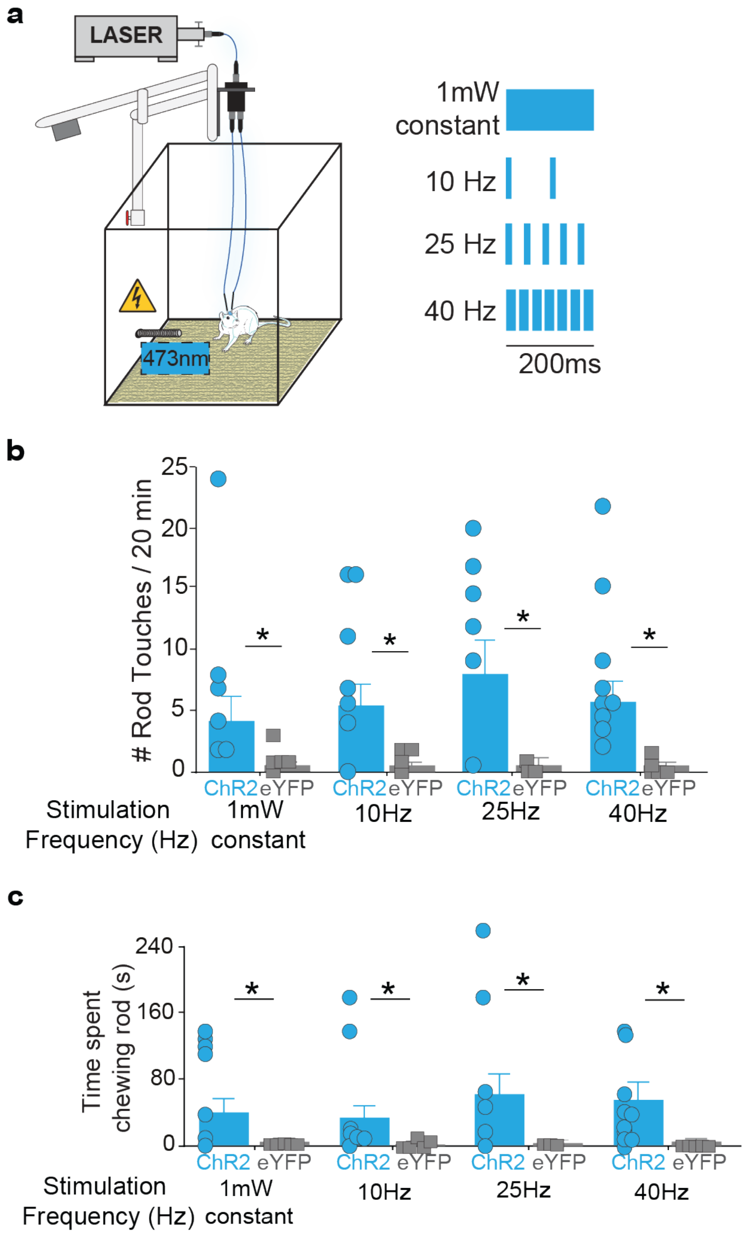

Supplementary Figure 5 CeA shock rod attraction occurs at multiple stimulation parameters (a) 4 separate test sessions, rats received different laser stimulation frequencies paired with shock rod interaction (473 nm; either constant light at 1 milliwatt (mW) intensity, or 10 hertz (Hz), 25 Hz, or 40 Hz at 10 mW intensity). **(b)** Shock rod touches during each laser frequency condition, ChR2 (*N=14*), eYFP (*N=6*), 2-way mixed ANOVA, frequency x virus interaction: $F_{2,48}$=1.38, p=.25; main effect of virus: $F_{1,48}$=3.48, p=.03; Dunnett 2-sided post-hoc t-tests ChR2 vs. eYFP, 1mW constant light: p=.04, 95%CI: 0.64, 7.8, *d*= 0.76; 10 Hz: p=.02, 95%CI: 0.9, 8.5, *d*= 1.12; 25 Hz: p=.02, 95%CI: 1.3, 13.5, *d*= 1.32; and 40 Hz: p=.01, 95%CI: 1.4, 8.7, *d*= 1.14. **(c)** Time spent chewing shock rod during each laser frequency condition, ChR2 (*N=14*), eYFP (*N=6*), 2-way mixed ANOVA, frequency x virus interaction: $F_{2,48}$=0.98, p=.45; main effect of virus: $F_{1,48}$=2.52, p=.04; Dunnett 2-sided post-hoc t-tests ChR2 vs. eYFP, 1mW constant light: p=.03, 95%CI: -294, -12, *d*= 1.10; 10 Hz: p=.03, 95%CI: -297, -14, *d*= 1.02; 25 Hz: p=.04, 95%CI: -306, -9, *d*= 0.92; and 40 Hz: p=.02, 95%CI: -293, -18; *d*= 1.14. Data represents mean and SEM. *p<.05

Shock Rod Condition	Fos+ Count (mean±SEM / 300µm x 300µm x 40µm)			ANOVA (F-statistic, p-value)		ChR2 vs. eYFP (Bonferroni adjusted p-value)
**Region**	**Homecage (N=4)**	**CeA ChR2 (N=9)**	**eYFP (N=7)**	**F**	***p***	***p***
Anterior Insula	6±.7	4.7±1.4	6.9±1.7	3.07	0.06	
Posterior Insula	2±0.9	18.9±4.3	7.1±1.3	4.28	0.02*	*0.03*
Infralimbic	1.5±0.3	8±1	5.6±1	4.38	0.02*	*0.012*
Prelimbic	0.5±0.3	4.6±1	3.1±1.3	1.72	0.20	
mOFC	1.25±0.5	7.1±0.1	4.3±2	1.59	0.04*	*0.03*
NAc Core	3.25±1.7	3.0±0.4	3.42±0.5	0.20	0.82	
Rostral NAc medial Shell	0.75±0.5	4.5±0.6	2.95±0.6	4.96	0.01*	*0.02*
Caudal NAc medial Shell	3.75±1.6	1±0.4	2.14±0.5	0.58	0.57	
Ventral Pallidum	3.5±1.3	3.8±0.78	2.8±0.52	0.57	0.57	
Dorsolateral Striatum	0.5±0.29	4.3±1.2	1.8±0.62	3.42	0.04*	*0.039*
Dorsomedial Striatum	0.5±0.30	3.3±0.63	2.6±1.04	1.33	0.28	
Perifo Lat Hypothalamus	2.5±0.6	6.2±0.7	3.5±0.49	3.13	0.05*	*0.014*
Lat Hypothalamus	4.5±1.2	2.5±1.0	3.6±0.95	2.33	0.11	
BLA	0.25±0.25	3.1±0.25	6±1.1	2.63	0.04*	*0.012*
BNST	0.4±0.5	3.7±1.4	8±1.2	2.68	0.03*	*0.05*
PVT	1.25±0.9	2.6±1	4.4±1.2	0.44	0.65	
Rostral VTA	0.5±0.3	2.3±0.5	2.0±0.75	1.02	0.37	
Caudal VTA	0.25±0.25	5.7±0.4	2.1±0.5	4.13	0.02*	*0.012*
Substantia Nigra	1.75±0.5	3.33±0.5	1±0.33	5.20	0.002**	*0.02*
PAG	1±0.6	1.3±.34	4.1±0.93	5.85	0.007**	*0.004*

Sucrose/Cocaine Condition	Fos+ Count (mean±SEM / 300µm x 300µm x 40µm)		ChR2 vs. Homecage Unpaired t-test (t-statistic, p-value)	
**Region**	**Homecage (N=4)**	**CeA ChR2 (N=4)**	**t**	***p***
Anterior Insula	0.5±1.3	2.1±1.9	1.2	*0.25*
Posterior Insula	2±.9	9.1±2.8	5.3	*0.006**
Infralimbic	1.25±.5	.38±.26	1.5	*0.16*
Prelimbic	0.5±.28	3.5±.8	2.5	*0.03**
mOFC	1.5±.29	4.5±1.4	2.15	*0.06*
NAc Core	0.8±.5	2±1.5	0.94	*0.37*
Rostral NAc medial Shell	0.75±.5	4.2±1.5	6.1	*0.004**
Caudal NAc medial Shell	3.25±1.7	3±1	1.26	*0.23*
Ventral Pallidum	3.5±1.3	4.6±.5	0.98	*0.35*
Dorsolateral Striatum	0.5±.29	2±.5	2.4	*0.03**
Dorsomedial Striatum	0.25±.25	1.12±.3	1.3	*0.21*
Perifo Lat Hypothalamus	2±.6	3.8±.7	2.1	*0.06*
Lat Hypothalamus	0.65±.5	1.5±.54	0.37	*0.72*
BLA	3.25±.25	0.25±.5	4.3	*0.001**
BNST	2±.4	0.2±.5	4.7	*0.002**
PVT	3.12±1.5	1.25±.9	0.59	*0.56*
VTA	0.25±.5	6.13±2.2	7.5	*0.002**
Substantia Nigra	1.75±.47	3±.53	1.7	*0.12*
PAG	1.5±.6	3±1.3	2.2	*0.05*

Supplementary Table 1. Brain-wide Fos activation in shock rod attraction or narrowly focused sucrose/cocaine pursuit. Table shows Fos+ protein quantification in mesocorticolimbic regions after final exposure to shock rod condition (above) or sucrose/cocaine choice task (below). Above: "Shock rod condition" shows Fos+ protein quantification in mesocorticolimbic regions (left columns),

ChR2 rats (*N=9*), eYFP rats (*N=7*), and baseline rats taken from homecage (*N=4*). Below: "Sucrose/Cocaine condition" shows Fos+ protein quantification, ChR2 rats (*N=4*), homecage rats (*N=4*). "Fos+ Count" reflects mean of each group at each site ± standard error (SEM). One-way ANOVA's were performed for above shock rod conditions followed by two-sided post-hoc t-tests between ChR2 and eYFP rats (Bonferroni-corrected). Two-sided Unpaired t-tests between ChR2 and homecage rats were performed in sucrose/cocaine condition. *p<.05, **p<.01
