# Supplementary material for: The central amygdala recruits mesocorticolimbic circuitry for pursuit of reward or pain
Source: Nat Commun. 2020 Jun 1;11:2716. doi: 10.1038/s41467-020-16407-1 (PMC7264246; doi:10.1038/s41467-020-16407-1)
Supplement: Supplementary file 2 — Peer Review File [file 41467_2020_16407_MOESM2_ESM.docx]

Peer Review Information

**Journal:** Nature Communications

**Manuscript title:** The central amygdala recruits mesocorticolimbic circuitry for pursuit of sugar, cocaine, or pain

**Corresponding author name(s):** Dr Shelley Warlow

**Editorial notes:**

**non**

**Reviewer comments & decisions:**

**Reviewer comments, first version:**

Reviewers' comments:

Reviewer #1 (Remarks to the Author):

This report by Warlow et al. follows up on previous work by this group on the motivation-enhancing effect of central amygdala (CeA) activation. The authors show that pairing optogenetic CeA stimulation with sucrose or cocaine reinforcement enhances choice of that reinforcer relative to the alternative, in line with previous work. The major findings reported here relate to how CeA stimulation modulates defensive behaviors induced by threat/fear learning. Whereas control rats develop typical avoidance and defensive treading behaviors to a metal rod paired with electric shock, rats for whom this pairing is accompanied by CeA stimulation did not develop defensive behaviors and instead acquired a paradoxical tendency to approach and interact with the rod, resulting in repeated avoidable shocks, and were even willing to overcome a barrier to engage in this behavior. They also exhibited enhanced motivation for an auditory cue that was paired with CeA stimulation and shock. Thorough analysis of shock-rod exposure revealed that CeA stimulation recruited the mesocorticolimbic system, consistent with evidence that dopamine receptor blockade disrupted rod approach. Control experiments suggest that CeA stimulation was not reinforcing in itself, which is important and distinguishes this stimulation from, for instance, direct stimulation of the dopamine system. Interestingly, separate rats undergoing standard fear conditioning with an uncontrollable foot shock exhibited evidence of enhanced defensive (freezing and avoidance) behavior, though this set of results was difficult to evaluate, as discussed below.

I think this is very interesting study that has the potential to significantly influence our understanding of the neural circuitry underlying motivated behavior, and potentially how pathological behaviors develop. The paradoxical shock rod-approach behavior described here is novel and compelling and will likely stimulate new research. While my overall assessment of the manuscript is positive, I do have a number of concerns, outlined below.

1) The dose of flupentixol seems quite high and the reference paper used to support the dose seems to be incorrect. The Extended Data Fig 5e suggests that flupentixol produced a considerable amount of immobility. These details suggest that flupentixol treatment may have simply disrupted motor behavior, as opposed to showing that CeA stimulation enhanced shock rod approach via a dopamine-dependent process.

2) There are inconsistences in description of the fear conditioning experiment. The main text implies that cue-shock pairings were accompanied by CeA stimulation and this is implied in the enhanced odor avoidance effect in ChR2 rats. But the schematic doesn’t make this clear, and this did not come across in my reading of the methods/figure caption. It is difficult to evaluate the authors’ claims without resolving this issue. It is suggested that CeA stimulation enhances acquisition of fear learning (freezing/avoidance) in this situation, which would be very interesting in light of the contrasting results found in the shock rod experiment. This seems to be generally supported by the odor avoidance results, but the freezing data are less clear. In that test, the CeA was stimulated on half of CS+ trials and seemed to enhanced expression of conditioned freezing. This effect was mostly evident on the first trial, which is notable because the level of freezing in this CeA-stimulated condition was ~60%, similar to levels on the first trial in eYFP rats. If all ChR2 rats were indeed given CeA stimulation during fear conditioning trials, then the critical question is whether they showed more freezing on unstimulated (no laser) trials at test, relative to eYFP rats. The data in panel B suggest that ChR2 rats showed a reduction or perhaps a delay in conditioned freezing, and that this effect was ameliorated by stimulating the CeA at test. Of course, it is also possible that the groups simply differed in their pattern of freezing over trials. This seems particularly likely if there was, in fact, no CeA stimulation during conditioning trials.

3) In general, the statistical power for the fear conditioning experiment is fairly low (n’s 5 and 8). This is a bigger issue if a group difference in acquisition of conditioned freezing is being assessed, as appears to be the case. Again, the effects here are fairly modest and trial-dependent, which also makes power a bigger concern.

4) Did CeA stimulation alter startle reactions to shock? This might be difficult to quantify during shock-rod tests because of differences in exposure time, though perhaps on the first trial. If CeA stimulation was given on fear conditioning trials, then this data would be interesting and important to consider.

5) Fig 3B presents a subset of cFos expression data, but for completeness, it would be good to include data for all regions in an Extended data table, for instance. The dorsolateral neostriatum and bed nucleus of the stria terminalis are discussed briefly in text but are not indicated in Fig 3B schematic (minor point).

6) I suggest the authors consider referring to central amygdala in the title, given that this is a defining element of the study.


Reviewer #2 (Remarks to the Author):

In this manuscript, Warlow and colleagues show that optical stimulation of the CeA can produce attraction toward both rewarding and punishing stimuli. Interestingly, they find that stimulation on its own is not itself reinforcing, nor does it produce attraction to neutral non-salient stimuli. These findings are novel and very interesting, but my enthusiasm for the work was diminished by the writing and framing of the manuscript, the lack of a clear hypothesis, and the minimal characterization of the main effect. Major issues:

1. More background information is needed. Both the introduction and discussion sections should describe what is known about CeA function, since it is not obvious how the present results advance our understanding of this structure.

2. The framing of the paper does a disservice to the data, as it emphasizes implications for human psychopathology (addiction and self-harm) even though the connections are weak. Is the data really relevant to “understanding the neural bases of … a child’s attraction toward an abusive caregiver”? Because of the issues detailed below, I encourage the authors to frame the manuscript more as an exploration of the parameters that give rise to the behavioural phenomena rather than suggesting that the data provides insight into addiction and self-harm.
a. It is not clear that eliciting a preference for sucrose over cocaine or vice versa represents some addictive aspect of CeA stimulation. Do animals work harder for reward and consume more of it when it is paired with CeA stimulation?
b. Addiction is characterized by compulsive drug-taking despite negative consequences, but here maladaptive attraction to the shock rod persists only in the presence of negative consequences. How are these comparable?
c. Self-harm is usually characterized as a coping mechanism. The authors provide no evidence that animals are coping with anything, nor that the shock-seeking behaviour recapitulates any of the cognitive aspects of self-harm.

3. Better characterization of the shock rod effect will offer greater insight into the significance of the phenomenon and the underlying psychological processes. One or more of these ideas should be explored:
a. How does the behavioral effect of CeA stimulation relate to the salience of the stimulus? Is there more or less behavioral attraction with high shock intensities (high salience)?
b. Is the effect of CeA stimulation dependent on the presence of an unconditioned stimulus? Could you promote attraction to a neutral but highly novel stimulus? Is it possible to enhance attraction to aversive stimuli of other modalities? What about non-harmful aversive tastants, like quinine?
c. Does altering the incentive salience of reward by changing the animals’ internal state affect the magnitude of CeA stimulation-induced attraction? For example, how does reward satiety affect the magnitude of the CeA-driven effect? Does this effect differ when it is associated with a water reward under differing conditions of satiety?

4. The authors decide not to show data relating to approach/touch/chewing behaviours directed to an inedible wooden block or a dummy no-shock rod. These data should be shown because they are critical controls for the results in Figure 2 (maladaptive attraction to shock).

5. Sample sizes are lacking in some cases, and the authors seem to use inappropriate statistical tests to compensate. For example, in Figure 1e, one of the control groups has a n of 2, and this “population” data is plotted with SEM (which is meaningless when the n = 2). Also, the corresponding experimental group is not directly compared, statistically with the control data. Instead, a 2-way ANOVA is used to compare 2 control groups across different experiments. I don’t understand the rationale here. The authors should draw the appropriate comparisons between experimental and control groups using a 2-way ANOVA to make statistical claims about the significance of their findings with respect to controls.

6. How context influences the effect of CeA stimulation is a key discussion point, yet the authors need to elaborate on what parameters they believe are relevant. What parameters shift the effects of the stimulation from amplifying fear to promoting approach. Is the relevant parameter controllable vs uncontrollable outcomes? If so, do uncontrollable rewards elicit avoidance/defensive behaviours? How does this hypothesis fit with the authors’ conception of incentive salience? When CeA stimulation “amplifies the incentive salience” of noxious stimuli, why does it increase exploration of the noxious stimulus in one case, and increase the fearful response to a noxious stimulus in another? If it increases the degree to which animals will attend to an unconditioned stimulus, why doesn’t CeA stimulation enhance avoidance of a shock rod?

7. Figures 1 and 3 are cluttered and would benefit from having fewer words / symbolic diagrams. For example, the use of sugar pellets and cocaine syringes as data points in Figure 1e obscures the data itself. It adds unnecessary symbols and, in the case of the syringe, produces confusion with the appearance of a possible horizontal error bar.

8. The language used throughout the manuscript obscures what the authors are trying to convey. For example:
a.‘Excessive attraction’ (pg 1 ln 10) – How do the authors define that?
b. ‘Intense and narrowly pursued’ (pg 9 ln 15, pg 12 ln 22-23, pg 13 ln 20, pg 16 ln 17) – Adjectives like this are not defined or supported with quantification. What does it mean for the pursuit of reward to be intense and narrowly focused, especially when animals experience fewer than 12 reward trials per session?
c. ‘magnified defensive conditioned reactions’ (pg 1 ln 19-20, pg 12 ln 2, pg 13 ln 13, pg 14 ln 20, pg 15 ln 18, pg 16 ln 20) – How do authors know that an existing motivation has been magnified? In Extended Data Fig. 6, eYFP controls show no conditioned place avoidance. In what sense has CeA stimulation magnified a non-existent effect?
d. ‘amplifies attraction towards an aversive shock rod’ (pg 17 ln 6, pg 21 ln 1) – Are the authors amplifying an existing attraction to the shock rod?
e. ‘positive attraction and negative fear’ (pg 13 ln 6, pg 15 ln 23, pg 17 ln 6) – These words are redundant.
f. ‘particular situations’ (pg 2 ln 19, pg 16 ln 14) – Which situations? This should be made more explicit in the introduction (uncontrollable vs controllable shock).
g. ‘The combination was hijacked here by individualized CeA ChR2 pairings’ (pg 16 ln 16) – It is unclear what this means.
h. The brain-wide fos data doesn’t really fit with Fig. 3, nor with the paper itself. It might be worth moving this data to the supplemental figure and replacing it with the auditory fear conditioning data or with data characterizing the boundary conditions for the effects of CeA stimulation.


Reviewer #3 (Remarks to the Author):

This is an interesting manuscript probing how optogenetic activation of undefined CeA neurons can alter 'pursuit' of various stimuli. This set of experiments adds to the body of data suggesting that stimulation of CeA can impact much more than 'aversive' behaviors. Overall, this is well done, however there are some concerns.


-Conceptually, one of the concerns is that these experiments represent manipulation of an undefined population of neurons in the CeA. This is potentially quite important as elegant work from Luthi and Li, among others, have found that different populations of neurons can have dramatically different roles in regulation of behavior. Layering on top of this, different outputs from the CeA can differentially impact behaviors as well. I realize that this is a limitation of the model organism, but I think that this should be more clearly addressed in the limitation.

-The authors suggest that stimulation of CeA does not elicit chewing behavior, this stands in contrast with the work of DeAruaja suggesting that this is a major behavior following stimulation of CeA. it would be useful to compare and contrast your data with his (was a cell paper)

-The CeA has been linked with regulation of pain, this is not discussed, seems important given that one potential interpretation is that stimulation of the cea is altering the ability of the shock to elicit pain. this would be perhaps important to test, or at least discuss.

-In the text, the authors refer to extended data, but provide no sample size. I would suggest that they move this data in to the body of the manuscript and provide sample size in the text.


Minor issues:

The language in the manuscript is a bit informal at time. For example, I would not use the time 'eagerly' to describe the reduced latency of the rat to do something. A more precise description is perhaps a bit appropos. In this same vein, the discussion is a bit extensive and some of the words 'irresistable' appear to be somewhat 'loaded' terms.

It is unclear why there is a picture of a squirrel in figure 2. remove as it is non relevant.

**Author rebuttal, first version:**

Authors’ Response to Reviewer Comments:

We thank the reviewers for their helpful comments. Overall, in response, we have tried to improve our presentation to clarify both experimental methods (i.e., fear conditioning experiment and dose of dopamine antagonist) and alternative interpretations of CeA-induced shock rod effects (i.e., possible analgesic effects or craniofacial motor stimulation). We have additionally tried to clarify language and conceptual framing. The entire discussion has been extensively revised with these aims in mind. We hope reviewers will find our revision to be improved.

Reviewer #1 (Remarks to the Author):

This report by Warlow et al. follows up on previous work by this group on the motivationenhancing effect of central amygdala (CeA) activation. The authors show that pairing optogenetic CeA stimulation with sucrose or cocaine reinforcement enhances choice of that reinforcer relative to the alternative, in line with previous work. The major findings reported here relate to how CeA stimulation modulates defensive behaviors induced by threat/fear learning. Whereas control rats develop typical avoidance and defensive treading behaviors to a metal rod paired with electric shock, rats for whom this pairing is accompanied by CeA stimulation did not develop defensive behaviors and instead acquired a paradoxical tendency to approach and interact with the rod, resulting in repeated avoidable shocks, and were even willing to overcome a barrier to engage in this behavior. They also exhibited enhanced motivation for an auditory cue that was paired with CeA stimulation and shock. Thorough analysis of shock-rod exposure revealed that CeA stimulation recruited the mesocorticolimbic system, consistent with evidence that dopamine receptor blockade disrupted rod approach. Control experiments suggest that CeA stimulation was not reinforcing in itself, which is important and distinguishes this stimulation from, for instance, direct stimulation of the dopamine system. Interestingly, separate rats undergoing standard fear conditioning with an uncontrollable foot shock exhibited evidence of enhanced defensive (freezing and avoidance) behavior, though this set of results was difficult to evaluate, as discussed below.

I think this is very interesting study that has the potential to significantly influence our understanding of the neural circuitry underlying motivated behavior, and potentially how pathological behaviors develop. The paradoxical shock rod-approach behavior described here is novel and compelling and will likely stimulate new research. While my overall assessment of the manuscript is positive, I do have a number of concerns, outlined below.

1) The dose of flupentixol seems quite high and the reference paper used to support the dose seems to be incorrect. The Extended Data Fig 5e suggests that flupentixol produced a considerable amount of immobility. These details suggest that flupentixol treatment may have simply disrupted motor behavior, as opposed to showing that CeA stimulation enhanced shock rod approach via a dopamine-dependent process.

*We agree that our flupentixol dose was high, and in retrospect agree we cannot rule out a simple disruption of motor behavior. Therefore, we have decided to remove the flupentixol experiment from the current paper, and to re-evaluate the effects of dopamine blockade in future. Warlow, Naffziger, & Berridge Reviewer Response 2 A more thorough exploration of the effects of low-doses of neuroleptic blockade on shock rod approach is planned.*

2) There are inconsistences in description of the fear conditioning experiment. The main text implies that cue-shock pairings were accompanied by CeA stimulation and this is implied in the enhanced odor avoidance effect in ChR2 rats. But the schematic doesn’t make this clear, and this did not come across in my reading of the methods/figure caption. It is difficult to evaluate the authors’ claims without resolving this issue. It is suggested that CeA stimulation enhances acquisition of fear learning (freezing/avoidance) in this situation, which would be very interesting in light of the contrasting results found in the shock rod experiment. This seems to be generally supported by the odor avoidance results, but the freezing data are less clear. In that test, the CeA was stimulated on half of CS+ trials and seemed to enhanced expression of conditioned freezing. This effect was mostly evident on the first trial, which is notable because the level of freezing in this CeA-stimulated condition was ~60%, similar to levels on the first trial in eYFP rats. If all ChR2 rats were indeed given CeA stimulation during fear conditioning trials, then the critical question is whether they showed more freezing on unstimulated (no laser) trials at test, relative to eYFP rats. The data in panel B suggest that ChR2 rats showed a reduction or perhaps a delay in conditioned freezing, and that this effect was ameliorated by stimulating the CeA at test. Of course, it is also possible that the groups simply differed in their pattern of freezing over trials. This seems particularly likely if there was, in fact, no CeA stimulation during conditioning trials.

*We apologize that our fear conditioning procedures were not described with sufficient clarity in our original paper. We have now clarified our description of fear conditioning in this revision. Indeed yes, the Reviewer is correct that CeA laser stimulation was always delivered during Pavlovian training for fear conditioning, being paired with each CS+ sound and UCS footshock presentation. Including CeA ChR2 laser during training was required in order that the laser-shock history match the training for sucrose, cocaine, and shock-rod experiments. In all those experiments, CeA laser stimulation was also always paired with the designated sucrose, cocaine or shock rod encounter during initial training.*

*The crucial question here was whether this similar training procedure could nonetheless produce an opposite motivational effect: that is, potentiate Pavlovian fear CRs in later tests, in contrast to potentiating desire for paired sucrose, cocaine or shock rod. The answer was yes. This demonstrated the valence flexibility of CeA ChR2 induced motivation, and influence of situational features in determining CeA ChR2 stimulation contribution to fear vs desire.*

*This result showing CeA ChR2-induced increases in defensive CRs also helped rule out the possibility that CeA stimulation generally induced analgesia (which would have reduced defensive CRs), relevant to interpreting shock-rod attraction. While we do not rule out a partial analgesia possibility (described in Discussion) our Pavlovian fear results indicate that CeA ChR2 stimulation does not simply make shock UCSs much less aversive.*

*Regarding acquisition vs retrieval/expression roles of CeA ChR2 stimulation in fear conditioning, we agree that evidence for acquisition enhancement comes only from the significant increase in CS odor avoidance (when laser was withheld during CS test), which had been induced by previous CeA activation during training (i.e., CeA ChR2 group showed greater avoidance than eYFP group).*

*Regarding expression enhancement of fear CRs, the within-subject comparison of freezing CR results indicates that CeA ChR2 laser activation during a subsequent CS+ test Warlow, Naffziger, & Berridge Reviewer Response 3 session additionally enhanced freezing expression over control freezing CR levels by the same ChR2 rats to CS+ in absence of laser. To assess this, in the freezing CR expression test, CeA laser was delivered during 5 of the 10 CS+ test presentations on test day, whereas the remaining counterbalanced 5 CS+ presentations lacked the laser (allowing within-subject comparison). No footshock UCS was given in that test. We now explain all this more clearly in our modified methods section (page 42, line 1) and in the figure schematic (now in main figure 6).*

*As the reviewer notes, there were also between-group individual differences in freezing propensity between ChR2 and eYFP groups: groups differed even in baseline freezing levels in absence of CS+ (ChR2 vs eYFP unpaired t-test: t11 =3.12, p=.01). Therefore in this revision, we have followed the practice of some Pavlovian fear conditioning amygdala studies in using percent-change-from baseline measures of CR when comparing between-group effects of amygdala manipulation group vs. control group (i.e., Fam et al, Psychoneuroendocrinology, 2018), to avoid the potential confound that baseline freezing levels can confound eventual freezing (see Jacobs, Cushman, & Fanselow, J Neurosci Methods 2010). We now present CRs as % elevation in freezing data above within-subject baselines to better express the effect of CS+ and its potential modulation by CeA laser stimulation (% change in freezing during CS+ relative to an individual’s own baseline of pre-CS+ freezing).*

*With this measure, our new analyses of freezing CRs confirm, using a two-way ANOVA with laser vs. nonlaser trials as a within subject factor and ChR2 vs. eYFP as between subject factor, a significant interaction between laser vs. nonlaser conditions and ChR2 vs eYFP groups (F1,11=5.07, p=.036). Subsequent post-hoc tests indicate that this difference came primarily from the ChR2 group specifically having higher freezing CRs on laser trials than nonlaser trials (ChR2 Laser freezing = 59+/-7% elevation over individual’s own baseline vs. ChR2 Nonlaser freezing = 37+/-4% elevation over baseline; Bonferroni-adjusted p=.035), whereas laser had no effect on freezing CRs of the eYFP group. This indicates that CeA ChR2 laser stimulation magnified the expression of a freezing CR specifically in ChR2 rats.*

3) In general, the statistical power for the fear conditioning experiment is fairly low (n’s 5 and 8). This is a bigger issue if a group difference in acquisition of conditioned freezing is being assessed, as appears to be the case. Again, the effects here are fairly modest and trial-dependent, which also makes power a bigger concern.

*Yes it is true that the Pavlovian fear groups were not large, though still sufficiently large to support a statistically significant CeA ChR2 enhancement of both freezing CRs (above) and CS+ odor avoidance. Of course, our primary purpose in the Pavlovian fear experiment was simply a) to provide a valence contrast to our major surprising result of shock-rod attraction (important for demonstrating valence flexibility of CeA ChR2 effects), b) to show that CeA induction of rod attraction was situation-dependent (important for interpreting factors determining valence roles of CeA circuitry) and c) that CeA stimulation does not simply reduce the aversive impact of electric shocks (important for interpretation of shock rod effects).*

*It probably is not a major surprise to readers that CeA stimulation can magnify conditioned fear CRs in a traditional Pavlovian paradigm. But it is interesting that it can do so in the same CeA ChR2 in which it caused attraction to laser-paired shock rod. We fully agree that further studies with larger groups will be needed to more fully map out important details of Warlow, Naffziger, & Berridge Reviewer Response 4 these effects, but we believe our present results are sufficient to support the conclusions we draw here.*

4) Did CeA stimulation alter startle reactions to shock? This might be difficult to quantify during shock-rod tests because of differences in exposure time, though perhaps on the first trial. If CeA stimulation was given on fear conditioning trials, then this data would be interesting and important to consider.

*We thank the reviewer for raising this startle issue. We have now analyzed the startle incidence among rats upon their first touches of the shock rod. We found that the probability of showing a withdrawal/startle response upon first touch was 94+/-0.06% among laser-stimulated ChR2 rats compared to 86+/-0.09% among eYFP rats (unpaired t-test: t30=0.823, p=0.42). This further suggests that CeA ChR2 stimulation did not reduce startle or simply nullify the impact of shock UCS (now included in results and in main figure 3A).*

5) Fig 3B presents a subset of cFos expression data, but for completeness, it would be good to include data for all regions in an Extended data table, for instance. The dorsolateral neostriatum and bed nucleus of the stria terminalis are discussed briefly in text but are not indicated in Fig 3B schematic (minor point).

*Thank you for suggesting this. We have now added Supplemental Table 1 to provide Fos expression data and test statistics for each brain region.*

6) I suggest the authors consider referring to central amygdala in the title, given that this is a defining element of the study.

*Thank you for the suggestion. We agree with your point, and have now made the title: Dangerous desires: Central amygdala recruits mesocorticolimbic circuitry for pursuit of sugar, cocaine, or pain.*

Reviewer #2 (Remarks to the Author):

In this manuscript, Warlow and colleagues show that optical stimulation of the CeA can produce attraction toward both rewarding and punishing stimuli. Interestingly, they find that stimulation on its own is not itself reinforcing, nor does it produce attraction to neutral non-salient stimuli. These findings are novel and very interesting, but my enthusiasm for the work was diminished by the writing and framing of the manuscript, the lack of a clear hypothesis, and the minimal characterization of the main effect. Major issues:

1. More background information is needed. Both the introduction and discussion sections should describe what is known about CeA function, since it is not obvious how the present results advance our understanding of this structure.

*Thank you for suggesting this. We now have added a brief description of CeA neuronal subtypes and circuitry function and more citations to the discussion (page 2, page 13). Warlow, Naffziger, & Berridge Reviewer Response 5*

2. The framing of the paper does a disservice to the data, as it emphasizes implications for human psychopathology (addiction and self-harm) even though the connections are weak. Is the data really relevant to “understanding the neural bases of … a child’s attraction toward an abusive caregiver”? Because of the issues detailed below, I encourage the authors to frame the manuscript more as an exploration of the parameters that give rise to the behavioural phenomena rather than suggesting that the data provides insight into addiction and self-harm. a. It is not clear that eliciting a preference for sucrose over cocaine or vice versa represents some addictive aspect of CeA stimulation. Do animals work harder for reward and consume more of it when it is paired with CeA stimulation?

*In this revision we now attempt to emphasize experimental explorations of the parameters, and have reduced mention of potential clinical implications throughout the paper. However, we expect that many readers may still want us to discuss potential clinical applications of these phenomena in a paragraph of the Discussion. We now point out that our shock rod, etc. results give a strong proof of principle demonstration that ‘wanting’ need not be proportional to target ‘liking’, and that the amygdala excitation interacting with mesocorticolimbic activation can focus motivation very narrowly on just the designated target. This is a basic point that is nonetheless very relevant to clinical processes postulated to occur in addicts by the incentivesensitization theory of addiction.*

*We now explicitly acknowledge that any consideration of potential applications is necessarily speculative, and have reduced most discussion of potential applications to paragraphs at end of discussion. We still believe readers may expect a brief discussion of what our conclusions could mean if applied apply to clinical topics, such as addiction and self-harm.*

*To answer the Reviewer’s specific question “Do animals work harder for reward and consume more of it when it is paired with CeA stimulation?”, yes, we have previously reported that rats reach higher breakpoints to earn sucrose or cocaine rewards when paired with CeA stimulation in breakpoint tests using progressive ratio schedules of effort demand (Robinson et al, 2014, Warlow et al, 2017). We now point this out (page 19, line 21), in addition to discussing the separate addiction criterion of persistence in face of adverse consequences.*

b. Addiction is characterized by compulsive drug-taking despite negative consequences, but here maladaptive attraction to the shock rod persists only in the presence of negative consequences. How are these comparable?

*We believe our results offer an expansion of the potential role of negative consequences in compulsive-appearing motivation. Traditionally, as the reviewer notes, animal models of addiction assume that adverse consequences must always be a negative-valence price paid to obtain a separate positive reinforcer. Willingness to pay that price is taken as a marker of compulsivity. Our results show that intuitive traditional interpretation may not capture all potential roles of adverse consequences when interacting with mesocorticolimbic circuitry. As the reviewer points out, our results show that an adverse consequence itself can enter into the target of incentive motivation, under particular mesocorticolimbic and situational conditions. Our results only provide a laboratory proof of principle demonstration. But once shown to exist, can such motivational mechanisms be ruled out in clinical addictions simply because traditional formulations don’t include them? We have now tried to clarify this in the discussion (page 13, line 9). Warlow, Naffziger, & Berridge Reviewer Response 6*

c. Self-harm is usually characterized as a coping mechanism. The authors provide no evidence that animals are coping with anything, nor that the shock-seeking behaviour recapitulates any of the cognitive aspects of self-harm.

*Yes, we agree that self-harm is usually characterized as a coping mechanism. Again, in our view, traditional coping explanation may persist more for lack of explanatory alternatives, rather than because of actual evidence for sufficient coping benefits to fully explain most selfharm behavior. Here we are offering an alternative explanatory mechanism: that amygdalamesocorticolimbic circuitry interaction, in particular circumstances, can produce direct ‘wanting’ for what hurts, without need for additional coping benefits. It may be that this could at least be a second potential explanation to some cases of self-harm. However, we have tried again to emphasize the necessarily speculative nature of discussing any potential clinical applications (page 20).*

3. Better characterization of the shock rod effect will offer greater insight into the significance of the phenomenon and the underlying psychological processes. One or more of these ideas should be explored:

a. How does the behavioral effect of CeA stimulation relate to the salience of the stimulus? Is there more or less behavioral attraction with high shock intensities (high salience)?

b. Is the effect of CeA stimulation dependent on the presence of an unconditioned stimulus? Could you promote attraction to a neutral but highly novel stimulus? Is it possible to enhance attraction to aversive stimuli of other modalities? What about non-harmful aversive tastants, like quinine?

c. Does altering the incentive salience of reward by changing the animals’ internal state affect the magnitude of CeA stimulation-induced attraction? For example, how does reward satiety affect the magnitude of the CeA-driven effect? Does this effect differ when it is associated with a water reward under differing conditions of satiety?

*We agree with the reviewer, and believe that each of these proposals is important. We do not have conclusive answers but hope to explore each of them more thoroughly in future studies. At present, we do have some relevant data, but these would need to be extended before taking a public position.*

*Regarding the question of (a), namely whether there is more behavioral attraction with higher shock rod intensities, we have pilot observations that suggest there is an inverted Ushaped curve relating intensity to attraction. That is, both very low and very high rod shock intensities appear to deter rod explorations, reducing laser pairings, and reducing attraction. Moderate shock intensities support CeA ChR2-induced rod attraction, though we point out this intensity is still strong enough to elicit withdrawal/startle reactions in all rats and to elicit motivated defensive treading/burying in most eYFP control rats. Thus our pilot results suggest there are intensity limits to to CeA-induced attraction, but more parametric studies would be needed to systematically assess this.*

*Regarding (b), namely, is having a simultaneous UCS an important component of CeA ChR2 attraction, yes, we think so, and now discuss this more clearly in the Discussion. The lack of a UCS may be why CeA ChR2 rats ignore an identical but unelectrified ‘dummy rod’ that offers similar CS properties without UCS shock, why they are not highly attracted to a laserpaired wooden block, and why CeA laser self-stimulation by touching a metal spout object is relatively weak (even missing completely for many rats here). Possibly using a more salient CS Warlow, Naffziger, & Berridge Reviewer Response 7 stimulus might increase its eligibility to become a target too, as the Reviewer suggests. Conceivably, for example, a more salient CS might help facilitate laser self-stimulation. Regarding the question of novelty, we do not know the answer, but since novelty would quickly fade across trials, the CS might need to be different each time. These issues remain to be explored. Also to be explored is whether a cue that predicts intraorally-delivered quinine paired with CeA stimulation would become attractive (for example, tested in an autoshaping task as in (Robinson et al, 2013).*

*Regarding (c), or whether changes in physiological states would modulate CeA ChR2 attraction, yes, we believe so. We predict that CeA ChR2 stimulation would interact with such internal states, since our hypothesis is that CeA ChR2 stimulation works by controlling incentive salience mechanisms which do interact with relevant physiological states. Also, here, shock rod attraction required continual CeA ChR2 excitation (decaying and replaced by defensive reactions in the no-laser test session), and within-session CeA excitation is a type of physiological state. We also note that defensive treading-burying behavior toward the shock rod is itself sensitive to changes in internal state (e.g., thirst and hunger; Saldivar Gonzalez et al, 1996; Davis et al, 1981a,b,c). Future experiments will be needed to systematically test effects of altering internal states, but we now mention the issue in discussion to acknowledge this important point (page 16).*

4. The authors decide not to show data relating to approach/touch/chewing behaviours directed to an inedible wooden block or a dummy no-shock rod. These data should be shown because they are critical controls for the results in Figure 2 (maladaptive attraction to shock).

*Yes, we agree, and thank the reviewer for this suggestion. We have now added data on chewing on inedible block and “dummy” no-shock rod to the results and figures (page 6 and Supplementary Figure S4).*

5. Sample sizes are lacking in some cases, and the authors seem to use inappropriate statistical tests to compensate. For example, in Figure 1e, one of the control groups has a n of 2, and this “population” data is plotted with SEM (which is meaningless when the n = 2). Also, the corresponding experimental group is not directly compared, statistically with the control data. Instead, a 2-way ANOVA is used to compare 2 control groups across different experiments. I don’t understand the rationale here. The authors should draw the appropriate comparisons between experimental and control groups using a 2-way ANOVA to make statistical claims about the significance of their findings with respect to controls.

*We have now additionally included individual data points to clarify the fact that population data alone makes SEMs uninterpretable for small groups (see Figure 2C). We believe we used statistically appropriate tests, but apologize for not adequately explaining what we did and for graphs that were confusing. For statistical tests, we performed a 2-way mixed betweensubjects/within-subjects ANOVA to compare laser and nonlaser responses between ChR2 and eYFP rats, which gave a significant effect of virus group (F1,10=4.6, p=.046, reflected in Figure 2C). All groups compared were in the same experiment, and so we now display both ChR2 and eYFP groups on the same graph to avoid confusion.*

6. How context influences the effect of CeA stimulation is a key discussion point, yet the authors need to elaborate on what parameters they believe are relevant. What parameters shift the effects Warlow, Naffziger, & Berridge Reviewer Response 8 of the stimulation from amplifying fear to promoting approach. Is the relevant parameter controllable vs uncontrollable outcomes? If so, do uncontrollable rewards elicit avoidance/defensive behaviours? How does this hypothesis fit with the authors’ conception of incentive salience? When CeA stimulation “amplifies the incentive salience” of noxious stimuli, why does it increase exploration of the noxious stimulus in one case, and increase the fearful response to a noxious stimulus in another? If it increases the degree to which animals will attend to an unconditioned stimulus, why doesn’t CeA stimulation enhance avoidance of a shock rod?

*To some extent, specifying the most crucial factors controlling the valence ‘switch’ is also a bit speculative at this point, because it would require parametric variations of one factor at a time. At present, we have distinct shock-rod/cocaine-sucrose situations vs. Pavlovian fear situation that switched valence between positive and negative. We also have several situations where CeA ChR2 remained relatively neutral (dummy no-shock rod; wood block; selfstimulation for most rats).*

*We can at least identify several potential factors involved in these situations that may control the switch, and we now try to discuss these candidates more clearly. For positive, we suggest important contributing factors may be 1) presence of either a reward UCS or 2) moderately intense electric shock UCS in shock-rod situation, combined with instrumental control over shock and presence of a safety zone, and presence of discrete source of shock that functions as a physical spatially-located cue that can be approached (i.e. the localized shock rod). Conversely, we suggest CeA ChR2 motivational valence may become negative when a shock UCS was uncontrollable and inescapable in the traditional Pavlovian fear situation, and not assignable to any discrete localized cue (i.e., the footshock came from everywhere in the chamber from the rat’s point of view, rather than from a localizable threat object). Possibly higher intensity of footshock than rod shock also contributes to CeA ChR2 negative valence in Pavlovian fear conditioning, though intensity comparisons may not need to be factor in that oral rod shocks often occurred on mouth. Future experiments would be needed to tease apart the relative contributions of these various factors to determining valence, but we have tried to at least to mention them all as potential contributors in the Discussion (page 19).*

7. Figures 1 and 3 are cluttered and would benefit from having fewer words / symbolic diagrams. For example, the use of sugar pellets and cocaine syringes as data points in Figure 1e obscures the data itself. It adds unnecessary symbols and, in the case of the syringe, produces confusion with the appearance of a possible horizontal error bar.

*Thank you for the suggestion. We have tried to clarify the figures. Figure 1 has been broken up into 2 separate figures to reduce clutter, and extraneous text has been removed (now Figures 1 and 2). Figure 3 has now been broken up into 2 figures (now Figures 4 and 5). Additionally, we now use fewer pellet/syringe icons and keep them only in cases where we think they help the viewer interpret the data.*

8. The language used throughout the manuscript obscures what the authors are trying to convey. For example:

a. ‘Excessive attraction’ (pg 1 ln 10) – How do the authors define that?

b. ‘Intense and narrowly pursued’ (pg 9 ln 15, pg 12 ln 22-23, pg 13 ln 20, pg 16 ln 17) – Adjectives like this are not defined or supported with quantification. What does it mean for the Warlow, Naffziger, & Berridge Reviewer Response 9 pursuit of reward to be intense and narrowly focused, especially when animals experience fewer than 12 reward trials per session?

c. ‘magnified defensive conditioned reactions’ (pg 1 ln 19-20, pg 12 ln 2, pg 13 ln 13, pg 14 ln 20, pg 15 ln 18, pg 16 ln 20) – How do authors know that an existing motivation has been magnified? In Extended Data Fig. 6, eYFP controls show no conditioned place avoidance. In what sense has CeA stimulation magnified a non-existent effect?

*We define excessive attraction operationally as CeA ChR2 laser-pairing attraction exceeding twice that of control eYFP levels of sucrose or cocaine attraction, or by repeated CeA ChR2 attraction to the normally-avoided shock rod (e.g., > 4 shocks per session), typically leading to repeated shocks in the same session. This seems excessive in our view, and maladaptive in the case of shock rod. We operationally define pursuit as also being narrow when >90% of all instrumental responses were directed toward obtaining one target (i.e. laser-paired sucrose or laser-paired cocaine) and < 10% of reponses are directed to the alternative target (i.e., equivalent sucrose or cocaine, without laser). We agree with the reviewer’s point that eYFP odor avoidance here was not actually multiplied from zero (although freezing CRs of ChR2 rats were magnified by laser). We now rephrase the statement as “potentiate defensive CRs” rather than “multiply defensive CRs”. We have also tried to clarify the above definitions in the manuscript.*

d. ‘amplifies attraction towards an aversive shock rod’ (pg 17, 21) – Are the authors amplifying an existing attraction to the shock rod?

*Yes, again we accept this point about multiplicative amplification, and now rephrase as “creates attraction toward an aversive shock rod.”*

e. ‘positive attraction and negative fear’ (pg 13, pg 15, pg 17) – These words are redundant.

*We suggest the terms are not necessarily redundant (e.g., people go on roller coasters for positively-valenced fear), though we agree fear and negative valence are semantically related and usually occur together. We have tried to clarify terms in the manuscript, and also removed the words positive and negative from several places where we agree they could be viewed as redundant.*

f. ‘particular situations’ (pg 2, 16) – Which situations? This should be made more explicit in the introduction (uncontrollable vs controllable shock).

*We now try to describe more explicitly situational factors such as uncontrollable vs. controllable, etc.,.*

g. ‘The combination was hijacked here by individualized CeA ChR2 pairings’ (pg 16) – It is unclear what this means.

*We agree, and have now rephrased the sentence.*

The brain-wide fos data doesn’t really fit with Fig. 3, nor with the paper itself. It might be worth moving this data to the supplemental figure and replacing it with the auditory fear conditioning data or with data characterizing the boundary conditions for the effects of CeA stimulation.

*We agree the auditory fear conditioning data are important, and have moved it to a main figure (now Figure 6). We still believe the brain-wide Fos data are important, because they show activation of mesocorticolimbic circuitry usually associated with incentive motivation (e.g., VTA, NAc) in CeA ChR2 rats attracted to the shock rod. It helps support the hypothesis that CeA activation really has induced positively-valenced appetitive motivation for the aversive shock rod. We therefore have moved it to its own separate figure for better graphical fit.*

Reviewer #3 (Remarks to the Author):

This is an interesting manuscript probing how optogenetic activation of undefined CeA neurons can alter 'pursuit' of various stimuli. This set of experiments adds to the body of data suggesting that stimulation of CeA can impact much more than 'aversive' behaviors. Overall, this is well done, however there are some concerns.

Conceptually, one of the concerns is that these experiments represent manipulation of an undefined population of neurons in the CeA. This is potentially quite important as elegant work from Luthi and Li, among others, have found that different populations of neurons can have dramatically different roles in regulation of behavior. Layering on top of this, different outputs from the CeA can differentially impact behaviors as well. I realize that this is a limitation of the model organism, but I think that this should be more clearly addressed in the limitation.

*Yes, we agree that specific cell-types within CeA, specific subregions of CeA, and specific output pathways have each been suggested to have specific valence roles in motivated behavior (i.e., Kim et al, 2017; Fadok et al, 2017; Haubensak et al, 2010; Ciocchi et al, 2010). Future studies will be needed in order to assign causal roles to particular neuronal subtypes or projections for CeA shock rod attraction. Our lab is now conducting CeA studies with CRF, D1, D2, and GAD Cre lines of transgenic rats, but results are still in progress. It is also conceivable that no single subtype/projection activation will match the full motivational power of our hSyn global activation here: that would mean multiple CeA subsystems act synergistically together. Either outcome will be of interest, and we have now expanded the discussion to acknowledge this issue (see page 13).*

The authors suggest that stimulation of CeA does not elicit chewing behavior, this stands in contrast with the work of deAruajo suggesting that this is a major behavior following stimulation of CeA. it would be useful to compare and contrast your data with his (was a cell paper)

*We agree the deAraujo group identified a specific CeA à brainstem pathway that induced chewing of wooden blocks, and predatory attack on crickets; we now cite that paper. Theirs was a selective activation of a specific CeAà parvocellular reticular formation pathway. It may be that this particular CeAà brainstem circuit in mice controls chewing movements and craniofacial motor muscles in a more hardwired motor fashion than our global hSyn CeA stimulation induced here. We do agree those amygdala brainstem projections may have motor effects on chewing, but the chewing we observed here seemed mostly restricted to motivated CS targets such as the shock rod, the moving metal sucrose levers, or metal cocaine portholes (Warlow et al, 2017). For example, chewing was not observed here on the dummy (unelectrified) rod, nor was much chewing directed toward the laser-paired wood block*

The CeA has been linked with regulation of pain, this is not discussed, seems important given that one potential interpretation is that stimulation of the Cea is altering the ability of the shock to elicit pain. this would be perhaps important to test, or at least discuss.

*We agree that CeA may participate in pain regulation, including analgesia (e.g., Wilson et al, 2019). We now note in Discussion that our observation of chewing the shock rod among CeA ChR2 rats might conceivably involve a partial analgesic effect (page 16). However, we also note our rats still invariably flinched reflexively to shocks and showed a withdrawal startle response as mentioned above with new analyses. Also, the dummy no-shock rod was never attractive, although it had complete analgesic status in a sense. Finally, and perhaps most important, CeA ChR2 stimulation increased fearful freezing CRs and defensive odor avoidance in the Pavlovian fear conditioning paradigm. Increased fear CRs is the opposite of what would be expected if CeA ChR2 stimulation simply produced footshock analgesia. Thus, we do not rule out a possible minor analgesia contribution to rod chewing, but conclude that it cannot explain our major attraction or valence switch effects.*

In the text, the authors refer to extended data, but provide no sample size. I would suggest that they move this data in to the body of the manuscript and provide sample size in the text.

*Thank you for the suggestion. We now display all data and statistical tests in figures (main and supplementary), and for those analyses not in figures, sample size and statistical tests are reported in the body of the manuscript.*

Minor issues:

The language in the manuscript is a bit informal at time. For example, I would not use the time 'eagerly' to describe the reduced latency of the rat to do something. A more precise description is perhaps a bit appropos. In this same vein, the discussion is a bit extensive and some of the words 'irresistable' appear to be somewhat 'loaded' terms.

*We have now removed ‘eagerly’ and replaced it with explicit description of faster latency to approach (page 4). We have removed irresistible on page 16.*

It is unclear why there is a picture of a squirrel in figure 2. remove as it is non relevant.

*The squirrel in the photo was emitting defensive treading/burying toward the rattlesnake, also in the photo, as example of the evolved anti-predator aspect of the defensive reaction. But the photo was too small for this to be clearly seen, so we have removed the photo and replaced with a drawing of anti-predator behavior to show the ethological relevance of defensive burying/treading.*

Conclusion

*We are grateful for all comments and suggestions of reviewers. We think that the resulting changes they prompted have strengthened the manuscript. We believe the phenomena described here are robust and intriguing enough that readers will be interested to know about them, even though we all agree that much still remains to be done. We hope you will agree.*

Sincerely, Shelley Warlow, Erin Naffziger, and Kent Berridge

**Reviewer comments, second version:**

REVIEWERS' COMMENTS:

Reviewer #1 (Remarks to the Author):

The authors have made extensive changes in response to reviewer comments and the manuscript is much improved. I do not have any remaining concerns.


Reviewer #2 (Remarks to the Author):

The manuscript is much improved. It describes a very interesting and novel phenomenon, which has the potential to significantly impact the field. I have no further comments.


Reviewer #3 (Remarks to the Author):

The authors have adequately addressed my concerns.

**Author rebuttal, second version:**

**Reviewer comments, third version:**

**Author rebuttal, third version:**
